# Distinct gut microbial compositional and functional changes associated with impaired inhibitory control in patients with cirrhosis

**DOI:** 10.1080/19490976.2021.1953247

**Published:** 2021-08-04

**Authors:** Jasmohan S Bajaj, Amirhossein Shamsaddini, Andrew Fagan, Sara McGeorge, Edith Gavis, Masoumeh Sikaroodi, Lisa A. Brenner, James B Wade, Patrick M Gillevet

**Affiliations:** a Division of Gastroenterology, Hepatology and Nutrition, Virginia Commonwealth University and Richmond VA Medical Center, Richmond, Virginia, USA; b Microbiome Analysis Center, George Mason University, Manassas, Virginia; c Departments of Physical Medicine and Rehabilitation, Psychiatry, & Neurology, VA Rocky Mountain Mental Illness Research Education and Clinical Center, Aurora, Colorado, and University of Colorado, Anschutz Medical Campus, Aurora, Colorado, USA; d Department of Psychiatry, Virginia Commonwealth University, Richmond, Virginia, USA

**Keywords:** Hepatic encephalopathy, response inhibition, addiction, enterococcus, fecal microbiota transplant

## Abstract

Most cirrhosis etiologies, such as alcohol, hepatitis C, and obesity, involve behavior that require the loss of inhibitory control. Once cirrhosis develops, patients can also develop cognitive impairment due to minimal hepatic encephalopathy (MHE). Both processes could have distinct imprints on the gut-liver-brain axis. Determine the impact of inhibitory control versus traditional cirrhosis-related cognitive performance on gut microbial composition and function. Outpatients with cirrhosis underwent two tests for MHE: inhibitory control test (MHEICT, computerized associated with response inhibition) and psychometric hepatic encephalopathy score (MHEPHES, paper-pencil HE-specific associated with subcortical impairment) along with stool collection for metagenomics. MHEICT/not, MHEPHES/not, and discordant (positive on one test but negative on the other) were analyzed for demographics, bacterial species, and gut-brain modules (GBM) using multi-variable analyses. Ninety-seven patients [47 (49%) MHEPHES, 76 (78%) MHEICT, 41 discordant] were enrolled. *MHEPHES/not*: Cirrhosis severity was worse in MHEPHES without differences in alpha/beta diversity on bacterial species or GBMs. Pathobionts (Enterobacteriaceae) and γ-amino-butryic acid (GABA) synthesis GBM were higher in MHEPHES. *MHEICT/not*: We found similar cirrhosis severity and metagenomic alpha/beta diversity in MHEICT versus not. However, alpha/beta diversity of GBMs were different in MHEICT versus No-MHE patients. *Alistipes ihumii, Prevotella copri*, *and Eubacterium* spp. were higher, while *Enterococcus* spp. were uniquely lower in MHEICT versus no-MHE and discordant comparisons. GBMs belonging to tryptophan, menaquinone, GABA, glutamate, and short-chain fatty acid synthesis were also unique to MHEICT. Gut microbial signature of impaired inhibitory control, which is associated with addictive disorders that can lead to cirrhosis, is distinct from cirrhosis-related cognitive impairment.

## Introduction

Recent research suggests that an altered gut–brain axis is associated with several conditions, such as depression, anxiety, Parkinson’s disease, cirrhosis, and hepatic encephalopathy (HE).^1^ In cirrhosis, the impact on the brain is multifactorial and includes liver disease and multiple co-morbid conditions, as well as etiologies of the cirrhosis (e.g., alcohol, hepatitis C, obesity, and diabetes).^2^ Therefore, cirrhosis and HE are a microcosm of several factors that can impact the gut-brain axis, where gut microbial manipulation can be used as therapy.^3^ However, cognitive impairment in cirrhosis can precede the confusional status of overt HE.^3^ This cognitive impairment or minimal HE (MHE) is an anamnestic form of mild cognitive impairment, which portends further complications and can impact survival.^3^ MHE can be measured using several strategies, such as paper-pencil or computerized tests.^4^ The paper-pencil psychometric hepatic encephalopathy score (PHES), which includes five tests evaluates psychomotor speed, cognitive flexibility, and accuracy, while the inhibitory control test (ICT) evaluates working memory and inhibitory control.^3^ Since these tests interrogate separate parts of the brain, the gut contribution to individual test performance may increase our understanding of the gut-brain axis changes as cirrhosis progresses.^5^ Prior 16SrRNA analyses have shown that microbial taxa differentially associate with specialized brain imaging changes and specific cognitive tests in MHE, but a deeper metagenomic evaluation of the microbiota that are associated with specific cognitive impairments are needed.^6–9^

Our aim was to determine the linkage between bacterial metagenomic composition and function with specific cognitive tasks in patients with cirrhosis.

## Methods:

Outpatients with cirrhosis and healthy controls were recruited from Virginia Commonwealth University and the Central Virginia Veterans Healthcare system after IRB approval. After informed consent, all subjects underwent PHES and ICT. All stool were collected in RNALater with DNA extraction using published techniques.

Clinical data for patients with cirrhosis pertaining to demographics, liver disease etiology, Model for End-stage Liver Disease (MELD) score, prior HE history, and current therapy with lactulose or rifaximin and cognitive analysis were collected.*PHES details*: This is a validated five test paper-pencil battery which tests visuo-motor coordination, psychomotor speed, and reaction time.^5^ It consists of the number connection test-A, number connection test B, digit symbol test, serial dotting test, and line tracing test (has two components: time and errors). Of these, a high raw score on digit symbol and low time for completion or errors in the remaining tests indicate normal cognition. Based on population control values, the standard deviations are calculated for each sub-test, and the total is added to give one value.^3^ A low score on the total PHES score indicates better performance.

*ICT details:^10^* ICT involves the presentation of several letters at 500-ms intervals.^11^ Interspersed within these letters are the letters X and Y. The subject is instructed to respond to every X and Y during the initial part of the training run, which establishes the prepotent response. In the latter part of the training run, the subject is instructed only to respond when X and Y are alternating (called targets) and inhibit responding when X and Y are not alternating (called lures). After the training run, 6 test runs, which last approximately 2 min each, are administered with a total of 40 lures, 212 targets, and 1728 random letters in between. At the end of the test, the lure and target response rates are automatically calculated. Lower lure response and higher target response indicate better performance.^11^ MHE on PHES and ICT were based on norms created for the Virginia population.^12^ We also determined concordant or discordant (negative on one and positive on the other versus vice-versa) performances on these tests.

**Stool Collection and analysis details**: Metagenomic DNA from fecal samples was extracted using the MO BIO PowerFecal DNA Isolation Kit (Qiagen) and stored in our repository at −80°C until the metagenomics analysis.^13^ Samples were processed in an automated, high throughput manner using the QiaCube DNA/RNA Purification System (Qiagen) with bead beating in 0.1 mm glass bead plates. Isolated DNA was quantified and normalized using the Quant-iT Picogreen dsDNA Assay Kit. Shotgun metagenomic libraries were prepared with a procedure adapted from the Nextera Library Prep Kit (Illumina). Libraries were subsequently pooled and assessed using the Agilent Bioanalyzer. Sequencing was performed on either an Illumina NextSeq 550 (1 x 150 bp, NextSeq 500/550 High Output v2 kit) or an Illumina NovaSeq 6000 (1 x 100 bp, NovaSeq 6000 S2 Reagent Kit). Metagenomic analysis: Reads were processed and annotated using the BoosterShot in-house pipeline.^13^ Bcl files were converted to fastq format using bcl2fastq (Illumina). Cutadapt ^14^ was used for adapter and quality (final Q-score > 20) trimming. Reads shorter than 50 bp were filtered out using cutadapt, and all reads were trimmed to 100 bp prior to downstream alignment and annotation. Quality sequences were then aligned at 97% identity to a curated database (Venti) containing all representative genomes in RefSeq ^15^ for bacteria and additional manually curated strains using the BURST optimal aligner.^16^ Ties in alignment were broken by minimizing the overall number of unique Operational Taxonomic Units (OTUs). For taxonomic assignment, each input sequence was assigned the lowest common ancestor, which was consistent across at least 80% of all reference sequences tied for best hit. Counts were normalized to the species-level average genome length. OTUs accounting for less than one millionth of all species-level genomic markers were discarded, as well as those with either less than 0.01% of their unique genome or less than 1% of the whole genome covered by reads in any sample. The Shannon index, Chao1 index and observed OTU alpha diversity metrics were calculated from count tables rarefied to 40,000 reads per sample using the vegan^17^ R package.

We first analyzed patients who were PHES positive compared to PHES negative in the entire population and then similar analysis for those who were ICT positive and negative. We then analyzed those who were discordant and those who were MHEICT positive versus those who negative on ICT and similarly for MHEPHES (Fig S1). We performed MAAslin2 analysis of patients including age, gender, diabetes, PPI use, prior HE, lactulose and rifaximin use, psychoactive medications, depression and anxiety with the bacterial species comparing patients MHEICT versus not, similarly for PHES and for discordant patients.^18^ Alpha diversity (richness, Shannon, and Simpson), and beta-diversity (PERMANOVA and PCoA) were performed using BiomMiner.^19^ Finally, similar analyses were performed based on gut-brain module (GBM) between MHEICT/not, MHEPHES/not and those who were discordant.^8^ GBMs assembly database is a metabolic reconstruction framework specific for translating shotgun metagenomic data into microbial neuroactive metabolic potential was constructed based on extensive literature and database (MetaCyc) review.^8^ A set of 56 GBMs was assembled, each corresponding to a process of synthesis or degradation of a neuroactive compound by the members of the gut microbiota. Module structure follows the Kyoto Encyclopedia of Genes and Genomes (KEGG) database syntax as previously constructed for the gut microbial metabolic food chain.^9^ GBM presence in the metagenome is defined with a detection threshold of at least 66% coverage to provide tolerance to miss-annotations, and missing data are incomplete(draft) genomes. GBM abundances were derived from an orthologue abundance table using Omixer-RPM version 1.0 (https://github.com/raeslab/omixer-rpm) by matching calculated KEGG IDs with GBMs database curated KEGG IDs.

## Results:

### Demographic comparisons:

Ninety-seven patients with cirrhosis were included (Table 1). Approximately, half of the patients had MHEPHES diagnosed per norms. These patients were more likely to be advanced in their cirrhosis, with a higher proportion of men with alcohol-related etiology and PPI use compared to those negative on PHES. On the other hand, no significant changes in demographics and cirrhosis characteristics were found in MHEICT versus no-MHE. The results of MHE were concordant in 56 patients (15 patients negative on both and 41 positive on both). However, discordance was seen in the remaining 41 patients (35 patients MHEICT but not on PHES and 6 MHEPHES positive and negative on ICT, Figure S1).

**Table 1.** Details of Patients with Minimal Hepatic Encephalopathy on Psychometric Hepatic Encephalopathy Score (PHES) and on Inhibitory Control test (ICT) (N = 97)

	MHE on PHES			MHE on ICT		
	No (n = 50)	Yes (n = 47)	P value	No (n = 21)	Yes (n = 76)	P value
Age	57.9 ± 6.9	62.3 ± 6.4	0.01	58.1 ± 8.2	60.6 ± 6.5	0.21
Gender	30 (60%)	42 (85%)	0.004	15 (71%)	57 (75%)	0.74
PPI	26 (52%)	34 (69%)	0.03	13 (62%)	47 (62%)	1.0
Diabetes	13 (26%)	18 (37%)	0.19	9 (43%)	22 (29%)	0.23
MELD score	9.6 ± 3.1	12.7 ± 3.5	<0.0001	10.8 ± 3.7	11.2 ± 3.6	0.66
Etiology (HCV/Alc/Both/ NASH &other)	15/13/15/7	14/26/4/3	0.02	10/8/5/2	19/31/14/8	0.41
Depression	9 (18%)	14 (29%)	0.14	5 (24%)	18 (24%)	0.53
Anxiety	3 (6%)	3 (4%)	0.9	1 (5%)	5 (6%)	1.0
SSRI	7 (14%)	9 (19%)	0.51	4 (20%)	12 (16%)	0.32
SNRI	2 (4%)	1 (2%)	0.58	2 (10%)	1 (1%)	0.10
Opioids	4 (8%)	2 (4%)	0.43	1 (5%)	5 (6%)	1.0
Benzodiazepines	4 (8%)	1 (2%)	0.16	1 (5%)	4 (5%)	1.0
Gabapentin	4 (8%)	3 (7%)	0.70	1 (5%)	6 (8%)	0.88
Prior HE	24 (48%)	36 (77%)	<0.0001	14 (67%)	46 (61%)	0.61
Lactulose	21 (42%)	36 (77%)	<0.0001	10 (48%)	49 (65%)	0.16
Rifaximin	12 (24%)	27 (57%)	0.001	8 (38%)	31 (41%)	0.81
**Individual tests**						
PHES subtests						
Number connection A (seconds)	31.6 ± 9.5	65.8 ± 21.9	<0.0001	40.6 ± 19.2	50.3 ± 24.8	0.06
Number connection B (seconds)	73.8 ± 19.7	193.0 ± 112.0	<0.0001	98.8 ± 76.6	141.0 ± 103.0	0.05
Digit Symbol (number)	59.2 ± 13.6	33.3 ± 10.7	<0.0001	54.8 ± 18.2	44.4 ± 17.2	0.03
Serial dotting (seconds)	56.6 ± 14.2	104.0 ± 44.2	<0.0001	64.5 ± 22.8	83.8 ± 42.9	0.008
Line tracing Errors (number)	33.1 ± 24.1	45.0 ± 31.7	0.04	32.2 ± 32.0	40.7 ± 27.5	0.28
Line tracing time (seconds)	79.6 ± 22.8	130.8 ± 68.5	<0.0001	98.8 ± 61.1	106.0 ± 55.3	0.63
Total PHES (low = good)	−0.6 ± 1.5	−9.1 ± 3.5	<0.0001	−2.7 ± 4.7	−5.3 ± 5.0	0.03
**ICT subtest**						
ICT Lures (number out of 40)	12.5 ± 9.5	17.3 ± 9.9	0.02	3.1 ± 1.6	18.1 ± 8.7	<0.0001
ICT Targets (%)	96.2 ± 6.1	86.1 ± 14.8	<0.0001	97.7 ± 2.5	89.5 ± 13.2	<0.0001
ICT Weighted lures (number)	14.2 ± 11.7	27.9 ± 22.5	<0.0001	3.3 ± 1.8	25.6 ± 18.7	<0.0001

### Bacterial species comparisons:

**Figure 1.**
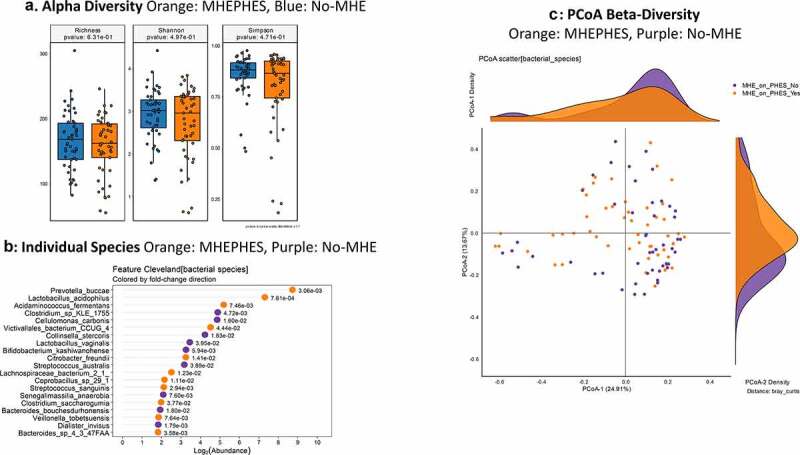
**Bacterial species comparison between patients with MHEPHES (n = 47) versus not (n = 50)**1A: Alpha diversity analyses did not show any differences between groups 1B: Cleveland plot derived from DESeq2 comparison 1 C: PCoA showing no significant separation between groups (PERMANOVA not significant)

**MHEPHES versus not**:. No changes in alpha/beta-diversity (PERMANOVA *p* = .23) were seen between PHES positive/negative patients (Figure 1). On MAAsLin2 top 30 variables with MHE-PHES, MELD score, several *Lactobacillus* spp. and species belonging to Enterobacteriaceae (*Klebsiella, Klyuvera, Pectobacterium)* but not *Enterococcus* spp. were higher. Several members of the Prevotellaceae family (*Prevotella ruminicola*), short-chain fatty acid (SCFA) producers such as Ruminococcaceae spp. and *Clostridium aerotolerans*, and species belonging to *Desulfovibrio* and *Bacteroides* were not associated with MHEPHES.

As a whole on MAasLin2, higher age and MELD score, and prior HE, lactulose and rifaximin use were associated with MHEPHES. Several species belonging to symbionts such as *Akkermansia*, as well as potential SCFA producers belonging to Lachnospiraceae and Ruminococcaceae were higher in those without MHE, while the reverse was seen with potential pathobionts belonging to Proteobacteria and urease-producing *Streptococcus* species (Table 2 and Table S1).

**Table 2.** MAAsLin2 Top 30 Bacterial Species and Clinical Variables associated with MHE on Individual tests

MHE on PHES	coefficient	P-value	Q-value	MHE on ICT	Coefficient	P-value	Q-value
*Clostridium_aerotolerans*	−7.3860288	1.14E-07	4.82E-05	*Pseudomonas_stutzeri*	−11.076275	6.59E-08	3.79E-06
*Ruminococcaceae_bacteriumCPB6*	−7.1714167	1.17E-06	0.00016485	*Clostridium_aerotolerans*	−8.5224458	6.89E-18	8.31E-15
*Hafnia_alvei*	−6.825657	6.92E-07	0.00012514	*Ruminococcaceae_bacterium_CPB6*	−8.1487537	2.94E-16	1.78E-13
*Desulfovibrio_fairfieldensis*	−6.559384	1.67E-06	0.00021139	*Enterococcus_*sp.*_HMSC064A12*	−7.351274	8.60E-16	3.46E-13
*Desulfovibrio_*sp.*_6_1_46AFAA*	−6.4232119	5.51E-06	0.0002792	*Enterococcus_*sp.*_HMSC070F12*	−6.9835319	7.14E-12	1.08E-09
*Ethanoligenens_harbinense*	−6.4218669	9.70E-06	0.00045469	*Enterococcus_*sp.*_HMSC076D08*	−6.8696824	5.83E-14	1.17E-11
*Hafnia_*sp.*_HMSC23F03*	−6.4054216	5.42E-06	0.0002792	*Enterococcus_*sp.*_HMSC035B04*	−6.7896399	1.10E-14	3.31E-12
*Prevotella_ruminicola*	−5.4842961	1.87E-07	5.93E-05	*Enterococcus_*sp.*_HMSC077E07*	−6.492291	3.67E-09	2.95E-07
*Corynebacterium_argentoratense*	−5.3129369	3.40E-06	0.00027784	*Ethanoligenens_harbinense*	−6.4231516	7.25E-10	7.29E-08
*Bacteroides_coprocola*	−2.898706679	1.38E-05	0.000581536	*Veillonella_seminalis*	−6.3948881	1.20E-10	1.45E-08
*Prevotellamassilia_timonensis*	−2.8492228	3.13E-06	0.00027784	*Desulfovibrio_*sp.*_6_1_46AFAA*	−6.2707191	5.57E-07	2.50E-05
**MELD score**	0.10135968	4.62E-06	0.0002792	*Enterococcus_*sp.*_HMSC060D09*	−6.245521	1.09E-08	7.72E-07
***Lactobacillus_zeae***	4.15539892	5.05E-06	0.0002792	*Clostridium_*sp.*_Marseille-P2415*	−6.1345706	1.49E-12	2.58E-10
***Prevotella_oris***	4.20943187	4.17E-06	0.00027784	*Enterococcus_*sp.*_HMSC073E08*	−6.045244	2.71E-10	2.98E-08
***Klebsiella_aerogenes***	4.27465841	3.71E-07	7.83E-05	*Enterococcus_*sp.*_HMSC077E04*	−5.9138986	1.12E-10	1.45E-08
***Synergistes_*sp.*_3_1_syn1***	4.53235446	5.18E-06	0.0002792	*Atopobium_minutum*	−5.7353621	2.91E-07	1.40E-05
***Cloacibacillus_evryensis***	4.8373667	5.82E-06	0.00028338	*Enterococcus_*sp.*_HMSC065H03*	−5.6796115	2.68E-09	2.31E-07
***Lactobacillus_acidophilus***	4.96799149	3.84E-08	4.23E-05	*Enterococcus_*sp.*_HMSC060E05*	−5.673559	9.69E-09	7.31E-07
***Lactobacillus_saerimneri***	5.035620618	1.26E-05	0.000549872	*Enterococcus_*sp.*_HMSC056C08*	−5.5581075	7.27E-08	3.99E-06
***Bifidobacterium_mongoliense***	5.05623943	2.42E-06	0.00027784	*Turicibacter_*sp.*_H121*	−5.3751102	7.76E-07	3.23E-05
***Lactobacillus_animalis***	6.40954148	3.53E-06	0.00027784	*Lactobacillus_mucosae*	−5.0214959	1.62E-09	1.51E-07
***Campylobacter_helveticus***	6.5153248	5.10E-06	0.0002792	*Enterococcus_faecium*	−4.9810595	1.60E-14	3.87E-12
***Kluyvera_ascorbata***	6.6757588	8.73E-07	0.00013808	*Enterococcus_*sp.*_HMSC034B11*	−4.9491816	2.86E-07	1.40E-05
***Lactobacillus_perolens***	6.7806971	1.05E-05	0.00047562	*Pseudopropionibacterium_propionicum*	−4.5684879	6.28E-08	3.79E-06
***Lactobacillus_harbinensis***	6.89835971	6.69E-08	4.23E-05	*Dysgonomonas_capnocytophagoides*	−4.2552989	5.70E-08	3.62E-06
***Pectobacterium_polaris***	7.31494204	2.90E-06	0.00027784	*Enterococcus gallinarum*	−3.8421406	1.99E-08	1.33E-06
***Providencia_rettgeri***	7.87775259	4.01E-06	0.00027784	*Parvimonas micra*	−3.8132837	1.08E-07	5.67E-06
***Atopobium_minutum***	12.7125871	3.16E-06	0.00027784	*Lactobacillus sakei*	−3.6981035	6.35E-07	2.74E-05
***Allofustis_seminis***	13.4373102	4.02E-06	0.00027784	*Clostridium hylemonae*	−3.2696316	1.62E-06	6.53E-05
***Clostridium_*sp. *BNL1100***	13.6372933	2.53E-07	6.41E-05	***Dakarella massiliensis***	6.08660083	5.60E-07	2.50E-05

**MHEICT versus not**: Similar to PHES, no changes in alpha-diversity was seen and PERMANOVA of borderline significance (*p* = .08; Figure 3) was seen for beta-diversity. On MAAsLin2, most bacterial species in the top 30 were negatively linked to MHEICT with the majority of these belonging to *Enterococcus, Veillonella, Clostridia* and Ruminococcacae spp. *Dakarella massiliensis* was the only microbe linked to MHE-ICT in the top 30 (Table 3 and Table S2). Remaining microbes associated with MHEICT were *Alistipes ihumii, Megasphaera massiliensis, Prevotella copri, Eubacterium* spp., and *Bifidobacterium adolescentis*.

**Table 3.** MAAsLin2 Top 30 Bacterial Species and Clinical Variables

MHEICT only (n = 35) versus MHEPHES only (n = 6)			MHEPHES only (n = 6) versus No MHEPHES (n = 50)			MHEICT only (n = 35) versuss NoMHEICT (n = 21)		
Feature	Higher in	P-value	Feature	Higher in	P-value	Feature	Higher in	P-value
*Turicibacter_*sp. *H121*	PHESMHE-NoICT	1.25E-16	*Dysgonomonas capnocytophagoides*	MHEPHES	9.75E-04	***Clostridium* sp. *ASF502***	**NoMHEICT**	**2.98E-04**
*Enterococcus_*sp. *HMSC064A12*	PHESMHE-NoICT	1.29E-13	*Lautropia mirabilis*	MHEPHES	0.001	***Clostridium* sp. *D5***	**NoMHEICT**	**8.96E-04**
*Enterococcus_*sp. *HMSC035B04*	PHESMHE-NoICT	2.61E-13	*Fusicatenibacter_*sp. *2789STDY5834925*	MHEPHES	0.002	***Lachnoclostridium* sp.*_YL32***	**NoMHEICT**	**0.003**
*Enterococcus_*sp. *HMSC14A10*	PHESMHE-NoICT	1.58E-11	*Rothia_*sp. *HMSC072B03*	MHEPHES	0.003	***Blautia schinkii***	**NoMHEICT**	**0.008**
*Citrobacter_freundii complex_*sp.*_CFNIH2*	PHESMHE-NoICT	1.53E-10	*Absielladolichum*	MHEPHES	0.005	*Dakarella massiliensis*	MHEICT	0.008
*Enterococcus_sp HMSC073E08*	PHESMHE-NoICT	7.24E-10	MELD score	MHEPHES	0.005	***Erysipelotrichaceae_bacterium_2_2_44A***	**NoMHEICT**	**0.009**
*Proteus_mirabilis*	PHESMHE-NoICT	2.31E-09	*Enterococcus* sp. *HMSC069A01*	MHEPHES	0.005	***Lactobacillus timonensis***	**NoMHEICT**	**0.014**
*Enterococcus_*sp. *HMSC065H03*	PHESMHE-NoICT	2.33E-09	*Rothia_mucilaginosa*	MHEPHES	0.006	***Streptococcus infantarius***	**NoMHEICT**	**0.015**
*Enterococcus_*sp. *HMSC076D08*	PHESMHE-NoICT	2.83E-09	Rifaximin use	MHEPHES	0.008	*Prevotella intermedia*	MHEICT	0.020
*Enterococcus_*sp. *HMSC070F12*	PHESMHE-NoICT	3.42E-09	*Lactococcus_piscium*	MHEPHES	0.010	***Lactobacillus reuteri***	**NoMHEICT**	**0.023**
*Enterococcus_*sp. *HMSC077E07*	PHESMHE-NoICT	4.00E-09	*Eisenbergiella_tayi*	MHEPHES	0.010	***Dysgonomonas capnocytophagoides***	**NoMHEICT**	**0.024**
*Enterococcus_*sp. *HMSC060E05*	PHESMHE-NoICT	1.27E-08	*Enterococcus* sp. *HMSC066C04*	MHEPHES	0.011	*Cellulomonas carbonis*	MHEICT	0.025
*Clostridium* sp. *BNL1100*	PHESMHE-NoICT	2.54E-08	*Lachnospiraceae bacterium 3_1_57FAA_CT1*	MHEPHES	0.011	***Bifidobacterium animalis***	**NoMHEICT**	**0.029**
*Enterococcus_*sp. *HMSC056C08*	PHESMHE-NoICT	2.69E-08	*Enterococcus* sp. *HMSC061C05*	MHEPHES	0.012	*Prevotella_bergensis*	MHEICT	0.032
*Enterococcus_*sp. *HMSC034B11*	PHESMHE-NoICT	4.17E-08	*Coprobacillus* sp. *29 1*	MHEPHES	0.012	***Enterococcus_*sp.*_HMSC035C10***	**NoMHEICT**	**0.033**
*Enterococcus gallinarum*	PHESMHE-NoICT	1.34E-07	*Enterococcus* sp. *10F3_DIV0382*	MHEPHES	0.013	*Prevotella lascolaii*	MHEICT	0.036
*Enterococcus_*sp. *HMSC077E04*	PHESMHE-NoICT	2.41E-07	Lactulose use	MHEPHES	0.014	***Streptococcus equinus***	**NoMHEICT**	**0.037**
*Enterococcus asini*	PHESMHE-NoICT	5.91E-07	*Enterococcus* sp. *HMSC058D07*	MHEPHES	0.015	*Lactobacillus rogosae*	MHEICT	0.039
*Streptococcus_*sp. *HMSC072D07*	PHESMHE-NoICT	6.32E-07	*Candidatus_Saccharibacteria_oral_taxon_TM7x*	MHEPHES	0.019	***Clostridium hylemonae***	**NoMHEICT**	**0.043**
*Eisenbergiella tayi*	PHESMHE-NoICT	1.56E-06	*Rothia_*sp. *HMSC062F03*	MHEPHES	0.021	***Megamonas_*sp.*_Calf98_2***	**NoMHEICT**	**0.044**
***Prevotella copri***	**ICTMHE-NoPHES**	**1.64E-06**	*Clostridium* sp. *D5*	MHEPHES	0.021	***Microvirgula aerodenitrificans***	**NoMHEICT**	**0.047**
*Clostridium_*sp.*_ASF502*	PHESMHE-NoICT	1.73E-06	*Enterococcus* sp. *5B3_DIV0040*	MHEPHES	0.021	***Hungatella hathewayi***	**NoMHEICT**	**0.047**
*Streptococcus_*sp. *HMSC076C08*	PHESMHE-NoICT	1.23E-05	*Enterococcus gilvus*	MHEPHES	0.022	*Bacteroides bouchesdurhonensis*	MHEICT	0.048
*Dysgonomonas_capnocytophagoides*	PHESMHE-NoICT	1.67E-05	*Streptococcus* sp. *UMB0029*	MHEPHES	0.023	*Prevotella albensis*	MHEICT	0.048
*Prevotella_*sp.*_Marseille_P4119*	PHESMHE-NoICT	2.27E-05	*Streptococcus* sp. *HPH0090*	MHEPHES	0.023	*Prevotella buccalis*	MHEICT	0.049
*Enterococcus_*sp. *HMSC072F02*	PHESMHE-NoICT	2.73E-05	*Bacteroides paurosaccharolyticus*	MHEPHES	0.023	*Desulfovibrio desulfuricans*	MHEICT	0.049
***Megasphaera massiliensis***	**ICTMHE-NoPHES**	**3.14E-05**	*Enterococcus* sp. *HMSC035B04*	MHEPHES	0.025	***Lactobacillus gastricus***	**NoMHEICT**	**0.050**
***Eggerthella_*sp. *YY7918***	**ICTMHE-NoPHES**	**3.58E-05**	Prior HE	MHEPHES	0.025	***Lachnospiraceae_bacterium_3_1***	**NoMHEICT**	**0.050**
***Alistipes ihumii***	**ICTMHE-NoPHES**	**3.83E-05**	*Streptococcus_*sp.*_NPS_308*	MHEPHES	0.026	*Prevotella copri*	MHEICT	0.050
*Prevotella oralis*	PHESMHE-NoICT	4.50E-05	*Enterococcus* sp. *HMSC070F12*	MHEPHES	0.028	***Enterococcus faecium***	**NoMHEICT**	**0.050**
Variables that are positively linked with MHE on ICT not PHES are in bold font			All variables were higher in those with MHEPHES only versus the rest			Variables that are positively linked without MHE on ICT are in bold font		

**Discordant**: No changes in alpha/beta-diversity were seen in MHEICT only versus MHEPHES only patients (Figure 5). On MAAsLin2, several *Enterococcus, Streptococcus* and pathobiont gram-negative species were higher in MHEPHES only patients while *Prevotella copri, Eggerthela* and *Alistipes* spp. were higher in MHEICT only patients in the top 30 (Table 3 and Table S3). **MHEICT only versus No MHEICT**: For the 35 patients impaired on ICT versus 21 with normal performance, we found lower *Enterococcus* spp. and higher *Prevotella* spp., *Dakarella massiliensis* and potential autochthonous species in those with MHEICT only (Table 3). **MHEPHES only versus No MHEPHES**: When we compared the 6 patients only impaired on PHES to the 50 patients who had normal performance on PHES, we found higher *Enterococcus, Streptococcus* spp. in MHEPHES only patients. Also, higher cirrhosis severity (higher MELD score, Prior HE, lactulose and rifaximin use) were higher in those with MHEPHES only patients compared to No MHEPHES patients (Table 3).

### Gut-brain modules:

**Figure 2.**
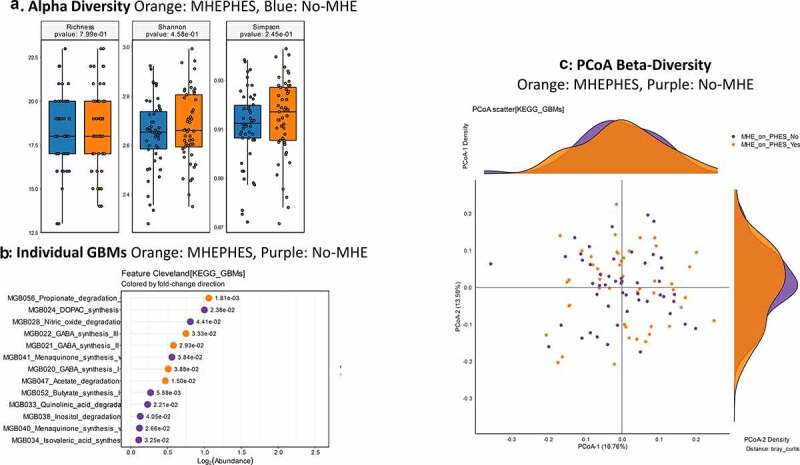
**Gut brain module comparison between patients with MHEPHES (n = 47) versus not (n = 50)** 1A: Alpha diversity analyses did not show any differences between groups 1B: Cleveland plot derived from Metastats comparison 1 C: PCoA showing no significant separation between groups (PERMANOVA not significant)

**MHEPHES versus not**: No differences in GBM alpha/beta-diversity was regardless of MHEPHES/not. MHEPHES patients had a higher abundance of GBMs related to GABA and glutamate synthesis, nitric oxide and propionate degradation on Metatstats, while those lower were related to butyrate, isovalerate, menaquinone and DOPAC synthesis, and degradation of quinolonic acid, NO and glutamate (Figure 2 and Table 4). **MHEICT versus not**: GBM alpha-diversity was higher in MHEICT versus No MHEICT, which also were separated on PCoA (PERMANOVA, *p* = .001). GBMs that were higher in MHEICT versus No MHEICT were focused on quinolonic acid, menaquinone, GABA, DOPAC and SCFA pathways, while the opposite was seen for GHB degradation (Figure 4 and Table 4). **Discordant**: In patients who were MHEICT but not PHES, there was higher GBM alpha-diversity. PERMANOVA (*p* = .001) also showed a clear separation between MHEICT-NoPHES and MHEPHES-NoICT (Figure 6 and Table 4). GABA synthesis III was the only GBM uniquely higher in MHEPHES. In MHEICT, DOPAC synthesis, SCFAs (Isovalerate synthesis-I KADH pathway, Butyrate synthesis-I), menaquinone synthesis, tryptophan and quinolinic acid synthesis, inositol and glutamate degradation and ClpB-ATP-dependent chaperone protein were higher.

**Table 4.** Comparison of Gut-Brain Modules Different in Patients According to Cognitive Strategy used

**PHES GBM Lineage**	LOG2FC	Direction	P-value
MGB056_Propionate_degradation_I	−1.06	MHE PHES	0.001806
MGB022_GABA_synthesis_III	−0.75	MHE PHES	0.03326
MGB021_GABA_synthesis_II	−0.58	MHE PHES	0.029268
MGB020_GABA_synthesis_I	−0.51	MHE PHES	0.038805
MGB047_Acetate_degradation	−0.47	MHE PHES	0.015038
MGB006_Glutamate_synthesis_I	−0.02	MHE PHES	0.011326
MGB027_Nitric_oxide_degradation_I_NO_dioxygenase	0	MHE PHES	0.012334
MGB029_ClpB_ATP_dependent_chaperone_protein	0.03	No MHE PHES	0.023788
MGB050_Glutamate_degradation_I	0.04	No MHE PHES	0.007327
MGB034_Isovaleric_acid_synthesis_I_KADH_pathway	0.11	No MHE PHES	0.032533
MGB040_Menaquinone_synthesis_vitamin_K2_I	0.12	No MHE PHES	0.026581
MGB038_Inositol_degradation	0.13	No MHE PHES	0.040531
MGB033_Quinolinic_acid_degradation	0.23	No MHE PHES	0.022053
MGB052_Butyrate_synthesis_I	0.27	No MHE PHES	0.005594
MGB041_Menaquinone_synthesis_vitamin_K2_II_alternative_pathway_futalosine_pathway	0.56	No MHE PHES	0.038444
MGB028_Nitric_oxide_degradation_II_NO_reductase	0.81	No MHE PHES	0.044083
MGB024_DOPAC_synthesis	1	No MHE PHES	0.023769
**ICT GBM Lineage**			
MGB032_Quinolinic_acid_synthesis	−5.1	MHE ICT	0.001
MGB043_Acetate_synthesis_I	−4.69	MHE ICT	0.005995
MGB047_Acetate_degradation	−3.97	MHE ICT	0.045925
MGB041_Menaquinone_synthesis_vitamin_K2_II_alternative_pathway_futalosine_pathway	−3.72	MHE ICT	0.02515
MGB033_Quinolinic_acid_degradation	−3.72	MHE ICT	0.0322
MGB040_Menaquinone_synthesis_vitamin_K2_I	−3.7	MHE ICT	0.00621
MGB034_Isovaleric_acid_synthesis_I_KADH_pathway	−3.54	MHE ICT	0.021862
MGB029_ClpB_ATP_dependent_chaperone_protein	−3.54	MHE ICT	0.029987
MGB006_Glutamate_synthesis_I	−3.37	MHE ICT	0.014197
MGB038_Inositol_degradation	−3.29	MHE ICT	3.97E-04
MGB053_Butyrate_synthesis_II	−3.25	MHE ICT	0.008637
MGB020_GABA_synthesis_I	−3.17	MHE ICT	0.008621
MGB024_DOPAC_synthesis	−3.17	MHE ICT	0.023021
MGB052_Butyrate_synthesis_I	−3.11	MHE ICT	0.007033
MGB050_Glutamate_degradation_I	−2.96	MHE ICT	0.042206
MGB021_GABA_synthesis_II	−2.81	MHE ICT	0.015873
MGB027_Nitric_oxide_degradation_I_NO_dioxygenase	−2.58	MHE ICT	0.002261
MGB056_Propionate_degradation_I	−2.21	MHE ICT	0.034031
MGB028_Nitric_oxide_degradation_II_NO_reductase	−1	MHE ICT	2.74E-05
**Discordant GBM Lineage**			
MGB005_Tryptophan_synthesis	6.15	ICTMHE-NoPHES	8.40E-05
MGB032_Quinolinic_acid_synthesis	5	ICTMHE-NoPHES	0.003334
MGB040_Menaquinone_synthesis_vitamin_K2_I	4.49	ICTMHE-NoPHES	0.009683
MGB033_Quinolinic_acid_degradation	4.43	ICTMHE-NoPHES	0.012096
MGB041_Menaquinone_synthesis_vitamin_K2_II_alternative_pathway_futalosine_pathway	4	ICTMHE-NoPHES	0.005421
MGB029_ClpB_ATP_dependent_chaperone_protein	4	ICTMHE-NoPHES	0.023231
MGB034_Isovaleric_acid_synthesis_I_KADH_pathway	3.91	ICTMHE-NoPHES	0.049365
MGB053_Butyrate_synthesis_II	3.81	ICTMHE-NoPHES	0.047532
MGB047_Acetate_degradation	3.7	ICTMHE-NoPHES	0.031842
MGB038_Inositol_degradation	3.17	ICTMHE-NoPHES	0.023127
MGB052_Butyrate_synthesis_I	3.09	ICTMHE-NoPHES	0.034837
MGB020_GABA_synthesis_I	2.81	ICTMHE-NoPHES	0.017337
MGB050_Glutamate_degradation_I	2.74	ICTMHE-NoPHES	0.017733
MGB044_Acetate_synthesis_II	2.68	ICTMHE-NoPHES	0.031584
MGB006_Glutamate_synthesis_I	2.66	ICTMHE-NoPHES	0.001651
MGB024_DOPAC_synthesis	1.58	ICTMHE-NoPHES	0.041681

**Figure 3.**
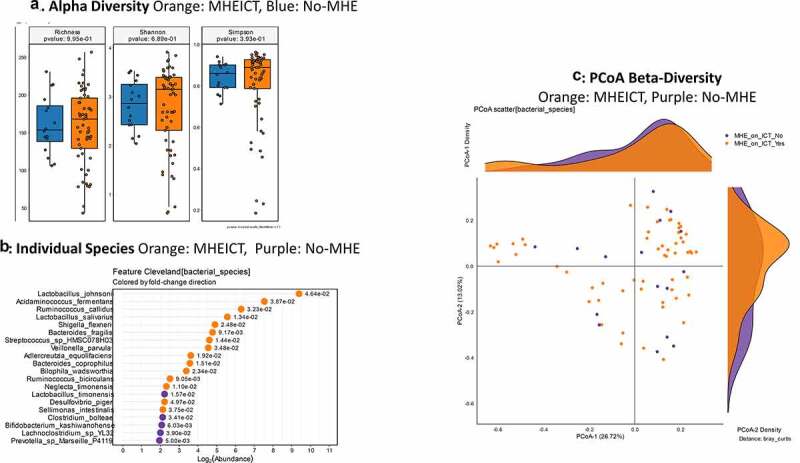
**Bacterial species comparison between patients with MHEICT (n = 76) versus not (n = 21)** 1A: Alpha diversity analyses did not show any differences between groups 1B: Cleveland plot derived from DESeq2 comparison 1 C: PCoA showing trend toward a significant separation between groups (PERMANOVA *p* = .08)

**Figure 4.**
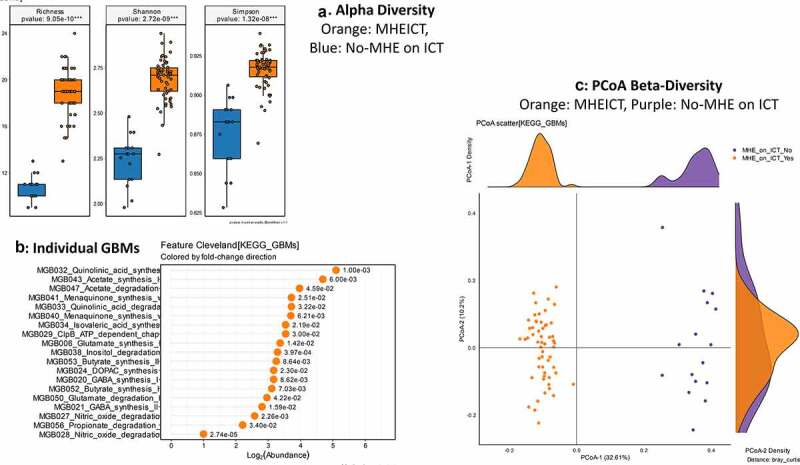
**Gut brain module comparison between patients with MHEICT (n = 76) versus not (n = 21)**1A: Alpha diversity analyses showed significantly higher diversity in the MHEICT group compared to no-MHE 1B: Cleveland plot derived from Metastats comparison 1 C: PCoA showing a significant separation between groups

**Figure 5.**
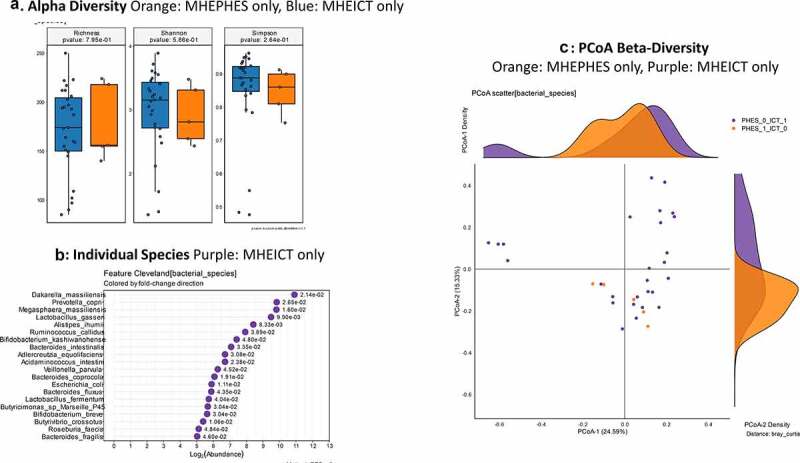
**Bacterial species comparison between patients with MHEICT-only (n = 35) versus MHEPHES only (n = 6)**1A: Alpha diversity analyses did not show any differences between groups 1B: Cleveland plot derived from DESeq2 comparison 1 C: PCoA showing no significant separation between groups

**Figure 6.**
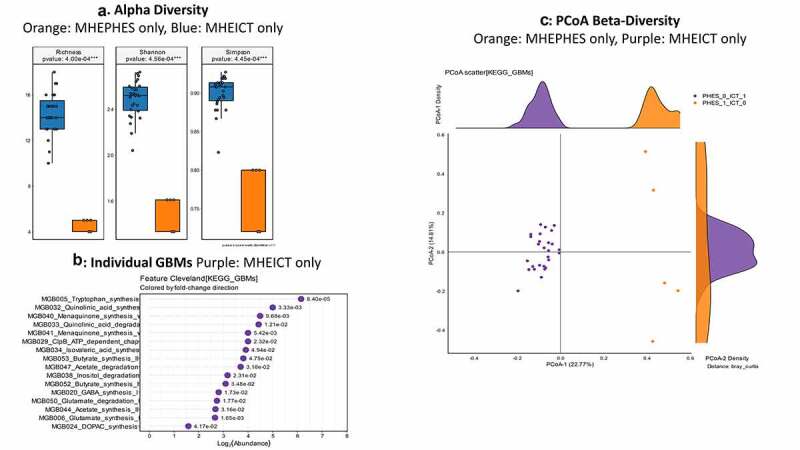
**Gut brain module comparison between patients with MHEICT-only (n = 35) versus MHEPHES only (n = 6)** 1A: Alpha diversity analyses showed significantly higher diversity in the MHEICT only group compared to MHEPHES only group 1B: Cleveland plot derived from Metastats comparison 1 C: PCoA showing a significant separation between groups

## Discussion:

In this study, we found that gut-brain axis changes in cirrhosis differed based on impairment on a measure of inhibitory control, a major determinant of several high-risk impulsive human behaviors which can result in self-harm. These include alcohol misuse, food addiction, and illicit drug use, which can lead to liver disease directly or through hepatitis C. With the further progression of liver disease, an inflammatory milieu and hyperammonemia can impair cognitive performance on visuospatial, psychomotor, and cognitive flexibility-related functions. Therefore, the cognitive impairment in cirrhosis could be a result of cirrhosis itself, the etiology of the cirrhosis or both. Gut-brain axis alterations in cirrhosis have been studied by several groups and favorable manipulation using laxatives, antibiotics, and fecal microbiota transplant have led to beneficial cognitive outcomes.^20–24^ A deeper characterization of functional microbial modules and their linkage with cognitive dysfunction in cirrhosis is relevant to define potential targets that can be extended beyond cirrhosis. As previously reported, there was relatively poor concordance between MHEICT versus MHEPHES.^25^ Regardless, we did not find alpha or beta-diversity changes between groups with/without both forms of MHE. This similarity is at odds with the demographic and cirrhosis profile since MHEPHES patients had more advanced liver disease compared to unimpaired patients while MHEICT patients did not. This points toward a relationship between the microbiota and cognitive function that could be independent of the liver disease.^26–31^

In addition, the PHES emphasizes psychomotor speed, which on a neuronal level reflects the integration between primary motor function (i.e., dopaminergic-based subcortical-cortical motor circuit) and non-motor function (i.e., cognition and emotion).^32^ It is a 5-test battery, which is mostly sub-cortical and is usually specific for cirrhosis. On the other hand, ICT evaluates working memory storage and an individual’s ability to override, or inhibit, a habitual behavioral response^33^ that depends on prefrontal cortex integrity. In addition to inhibitory control, good ICT performance requires strong working memory skills. Working memory (WM) involves the temporary storage, and subsequent manipulation of data. Given the PHES’s emphasis is on simple psychomotor speed (which depends on subcortical structures), compared to the more complex, and cognitively demanding task in ICT that emphasizes higher cortical processing, it is not surprising that more patients were impaired on ICT.

However, regardless of testing strategy, SCFA producers and symbionts tended to be lower in those with MHE even on multi-variable analyses. On the other hand, potential gram-negative pathobionts belonging to Enterobacteriaceae, as well as *Lactobacillus* and *Veillonella* spp. were higher in both MHE groups. These taxa have been associated with cirrhosis progression, as well as the production of GABA, which can promulgate cognitive impairment.^34^ This was further reflected in the GBMs with those centered around the synthesis of GABA, and the degradation of propionate and NO (through NO dioxygenase) were higher in MHE regardless of modality. GABA is a major inhibitory neurotransmitter, which is associated with cirrhosis progression and HE. GABA is produced by several different microbiota including *Lactobacillus, Escherichia*, and *Bifidobacterium*, which are elevated in advancing cirrhosis and in MHE patients.^35^ GABA synthesis I–II are over-represented in *E. coli* and *K. aerogenes* spp. and involve putrescine to GABA conversion via glutamate or 2-oxoglutarate.^36^ Glutamate is a major excitatory neurotransmitter while GABA is inhibitory; pathways overexpressing conversion to GABA are likely related to cognitive impairment. NO dioxygenase degradation usually protects against nitrosative stress through the expression of flavoHgb in bacteria and fungi,^37^ however, the overexpression can lead to oxidative stress, which has been found in HE.^38^ Moreover, the major inducer, NO, is suppressed in patients with cirrhosis and portal hypertension.^39^ Propionate is a major SCFA which can affect the gut barrier function as well as promote immune surveillance, neuronal health and integrity of the blood–brain barrier.^40^ Therefore, the association of MHE with enhanced propionate degradation is not surprising.

While MHE on PHES was associated with advancing cirrhosis severity, MHEICT was not. Therefore, the discordance between Enterobacteriaceae and *Enterococcus* spp. based on mode for MHE diagnosis is interesting because prior studies have indicated that both the taxa increase with advancing cirrhosis. Since this pattern of higher Enterobacteriaceae members in both MHE groups but reduction in *Enterococcus* abundance in MHEICT persisted despite multi-variable adjustment, it could reflect an underlying difference in gut-brain axis alteration independent of cirrhosis severity. The mechanism is unclear but serotonin, which is a product of *Enterococcus* spp.^34^ promotes inhibitory control and lowers impulsivity, which could be contributory to lower *Enterococcus* spp. in MHEICT.^41,42^ This higher *Enterococcus* spp. in MHEPHES and lower in MHEICT was further confirmed when subgroups impaired on only one test were compared to those that were unimpaired. In addition, similar patterns to that seen when the entire group of cognitively impaired patients were compared to the rest were also seen when exclusively impaired patients were analyzed. This included a greater role of cirrhosis severity in MHEPHES and higher *Prevotella* and *Dakarella* spp. in MHEICT. Moreover, since the prevalence of psychoactive medications and diagnoses were similar across groups, this is unlikely to be an epiphenomenon of medication use. Certain *Enterococcus* spp. are also able to synthesize dopamine, which is associated with lower impulsivity; therefore, lower *Enterococcus* may be contributory to poor response inhibition.^43–45^ This was further corroborated by higher DOPAC, a dopamine degradation metabolite, in MHEICT using GBM analysis.

Species associated with MHEICT but not PHES were *Prevotella* spp.,*Dakarella massiliensis, Megasphaera massiliensis*, and *Alistipes ihumii*. Two taxa, *Prevotella copri* and *Dakarella massiliensis (*member of Sutterellaceae) are associated with inflammation, altered glycemic status, and advancing liver disease, respectively.^46,47^ However, the other two species, *Megasphaera massiliensis* is a butyrate and medium chain FA producer ^48^ and *Alistipes* spp., associated with protection from disease progression in HE.^49^ This could reflect the similar cirrhosis severity between groups with/without MHEICT rather than MHEPHES. This was further extended by findings that clinical variables such as MELD score, HE, lactulose and rifaximin use were only significantly independent of bacteria in MHEPHES multi-variable analysis but not in MHEICT. This indicates that the microbial outputs could be more related to ICT rather than the cirrhosis severity, while PHES performance is the reverse. While these may be partly due to the relatively preserved hepatic function in MHEICT patients versus MHEPHES ones compared to unimpaired, these taxa were significant on MAASLin2 despite controlling for these clinical factors.

The only GBM higher in MHEPHES was GABA production through the shunt, which is higher in lactic acid-producing bacteria,^50^ which reflect worsened disease in cirrhosis. Despite the relatively similarity in cirrhosis severity, MHEICT patients had several uniquely higher GBM abundances than MHEPHES. Even more so than the bacterial species, several GBMs were lower in MHEPHES and higher in MHEICT that skewed toward several important processes. These included SCFA/branched-chain SCFA production, inositol, and glutamate degradation, DOPAC, tryptophan, menaquinone, and quinolinic acid synthesis and ClpB-ATP-dependent chaperone protein. Glutamate degradation through an NAD-linked dehydrogenase is ubiquitous and is ammoniagenic.^9^ Quinolinic acid degradation from aspartate is involved in the formation of NAD that is required for the above-mentioned glutamate degradation.^51^ Two pathways requiring chorismate resulting in tryptophan and menaquinone generation were higher in MHEICT, both of which are associated with neuroactive potential. Menaquinone is critical for electronic transport chain integrity and can be anti-oxidant in the brain.^52,53^ DOPAC is a degradation product of dopamine, which along with isovaleric acid, an SCFA, is associated with lower depression in the general population. Both isovalerate and DOPAC pathways were higher in MHEICT but not PHES patients. DOPAC production through isoflavinoids (quercetin) can be beneficial from a free radical scavenger perspective and is found in some human fecal bacteria, such as *Clostridium perfringens* and *Bacteroides fragilis*, but not *Escherichia coli* or *Lactobacillus acidophilus*. This fits the profile of cirrhosis progression associated with higher Lactobacillus and Enterobacteriaceae members and MHEPHES in contrast to ICT. Inositol degradation may be potentially injurious due to reduction in membrane stabilizing phosphatidylinositol and brain osmotic protector myoinositol and could potentiate ICT-related cognitive impairment. ClpB chaperone protein caseinolytic protease B (ClpB), which is found in Rikenellaceae and Clostridiaceae and are negatively related to obesity.^54^

These findings underline the association of metagenomic structural and functional changes in gut microbiota with differing cognitive profiles in patients with cirrhosis. Understanding cirrhosis as a culmination of etiologies and concomitant comorbid conditions is important to interpret these results and potentially extend them beyond this population. It is striking that despite minimal changes in cirrhosis severity between groups impaired on ICT, there was a major change in GBMs that was unique to this impairment compared to the traditional PHES modality while PHES changes largely followed the underlying cirrhosis itself. Unlike in the general population, we did not find a major impact of depression, anxiety, or other psychoactive medication use on either MHEICT or MHEPHES. This could be due to the major impact of cirrhosis on the microbiota that would reduce the relative influence of these medications or conditions. These unique changes to ICT could be due to reduced inhibitory control that is inherent in addictive disorders or substance abuse disorders that often precede cirrhosis. ICT-related changes focused on aromatic amino acid and SCFA metabolism suggest that microbiota involved in these pathways could specifically be targeted to improve outcomes. In prior studies, fecal microbiota transplant has been used to beneficially improve cognitive function in cirrhosis, and reduced craving toward alcohol.^55–57^ However, those studies were focused on one donor for all recipients. These results could form the basis for developing new therapeutic options focused on microbiota that are associated with inhibitory control changes that could be extended beyond cirrhosis.

Our study is limited by the relatively modest sample size and the large proportion who were positive on MHEICT. However, we analyzed discordance and found similar changes regardless of PHES impairment. We excluded those with active substance abuse to avoid confounding and had a relatively narrow age range with mostly men. Future studies across genders need to be performed.

We conclude that impairment on inhibitory control has a distinct metagenomic and GBM signature in the gut microbiota of patients with cirrhosis, which is independent of degree of cirrhosis severity, mood disorders, or psychoactive medications. This is different from microbial changes found with traditional psychometric hepatic encephalopathy score impairment that largely follows cirrhosis severity. Since impaired inhibitory control forms a major basis of addictive disorders, the microbial changes that are unique to this present an opportunity to design trials focused on manipulating these specific microbial taxa.

High scores on ICT targets and digit symbol indicate better performance, while low scores on all others, including composite PHES score, indicate better performance, MHE on PHES or ICT is adjusted for age, gender, and educational performance; the raw scores are presented above. Both indicates Hepatitis and alcohol.

Variables that are positively linked to MHE are in bold font, rest are associated with the absence of MHELOG2FC: Log 2-fold change.

## Supplementary Material

Supplemental Material
