# Supplementary material for: Distinct gut microbial compositional and functional changes associated with impaired inhibitory control in patients with cirrhosis
Source: Gut Microbes. 2021 Aug 4;13(1):1953247. doi: 10.1080/19490976.2021.1953247 (PMC8344770; doi:10.1080/19490976.2021.1953247)
Supplement: Supplemental Material [file KGMI_A_1953247_SM3660.zip › supplementary/Supplementary Information 6-3-21.docx]

**Supplementary Data Contents**

**Supplementary figure 1 legend**: Flow of patients and analyses of microbiota**.**

All 97 patients were tested with PHES and ICT. Of these, 47 were MHE on PHES and 50 were no-MHE on PHES. When ICT was used, 76 were MHEICT and 21 were No-MHE on ICT. 41 patients did and 15 did not have MHE on both testing strategies. Six patients were impaired only on PHES while 35 were impaired only on ICT. Comparisons performed are shown in the blue boxes, Red boxes are the number of patients who were impaired on that particular testing strategy. MHE: minimal hepatic encephalopathy, PHES: psychometric hepatic encephalopathy score, ICT: inhibitory control test.

**Supplementary tables 1-3:** MAAsLin2 analyses of bacterial species

**Table S1: MAASlin2 analysis of all MHE-PHES versus not bacterial species**

| **Feature** | **Higher in** | **Coefficient** | **P-Value** | **Q-Value** |
| --- | --- | --- | --- | --- |
| *Lactobacillus_acidophilus* | MHE_on_PHES_Yes | 4.973051 | 3.91E-08 | 4.03E-05 |
| *Lactobacillus_harbinensis* | MHE_on_PHES_Yes | 6.900241 | 6.68E-08 | 4.03E-05 |
| *Clostridium_aerotolerans* | MHE_on_PHES_No | 7.391607 | 1.12E-07 | 4.51E-05 |
| *Prevotella_ruminicola* | MHE_on_PHES_No | 5.484488 | 1.87E-07 | 5.65E-05 |
| *Clostridium_sp._BNL1100* | MHE_on_PHES_Yes | 13.63733 | 2.53E-07 | 6.11E-05 |
| *Klebsiella_aerogenes* | MHE_on_PHES_Yes | 4.283826 | 3.70E-07 | 7.44E-05 |
| *Hafnia_alvei* | MHE_on_PHES_No | 6.875143 | 5.93E-07 | 1.02E-04 |
| *Kluyvera_ascorbata* | MHE_on_PHES_Yes | 6.706016 | 7.93E-07 | 1.19E-04 |
| *Ruminococcaceae_bacterium_CPB6* | MHE_on_PHES_No | 7.17294 | 1.17E-06 | 1.56E-04 |
| *Desulfovibrio_fairfieldensis* | MHE_on_PHES_No | 6.564204 | 1.65E-06 | 1.98E-04 |
| *Bifidobacterium_mongoliense* | MHE_on_PHES_Yes | 5.056246 | 2.42E-06 | 2.58E-04 |
| *Prevotellamassilia_timonensis* | MHE_on_PHES_No | 2.870611 | 2.90E-06 | 2.58E-04 |
| *Pectobacterium_polaris* | MHE_on_PHES_Yes | 7.315085 | 2.90E-06 | 2.58E-04 |
| *Atopobium_minutum* | MHE_on_PHES_Yes | 12.7126 | 3.16E-06 | 2.58E-04 |
| *Corynebacterium_argentoratense* | MHE_on_PHES_No | 5.313152 | 3.40E-06 | 2.58E-04 |
| *Lactobacillus_animalis* | MHE_on_PHES_Yes | 6.419587 | 3.43E-06 | 2.58E-04 |
| *Providencia_rettgeri* | MHE_on_PHES_Yes | 7.877903 | 4.01E-06 | 2.65E-04 |
| *Allofustis_seminis* | MHE_on_PHES_Yes | 13.43734 | 4.02E-06 | 2.65E-04 |
| *Prevotella_oris* | MHE_on_PHES_Yes | 4.209948 | 4.17E-06 | 2.65E-04 |
| *Lactobacillus_zeae* | MHE_on_PHES_Yes | 4.155405 | 5.05E-06 | 2.65E-04 |
| *Campylobacter_helveticus* | MHE_on_PHES_Yes | 6.516376 | 5.09E-06 | 2.65E-04 |
| *Synergistes_sp._3_1_syn1* | MHE_on_PHES_Yes | 4.532599 | 5.18E-06 | 2.65E-04 |
| **MELD score** | MHE_on_PHES_Yes | 0.266079 | 5.30E-06 | 2.65E-04 |
| *Hafnia_sp._HMSC23F03* | MHE_on_PHES_No | 6.406445 | 5.41E-06 | 2.65E-04 |
| *Desulfovibrio_sp._6_1_46AFAA* | MHE_on_PHES_No | 6.42446 | 5.49E-06 | 2.65E-04 |
| *Cloacibacillus_evryensis* | MHE_on_PHES_Yes | 4.837626 | 5.82E-06 | 2.70E-04 |
| *Ethanoligenens_harbinense* | MHE_on_PHES_No | 6.422358 | 9.68E-06 | 4.33E-04 |
| *Lactobacillus_perolens* | MHE_on_PHES_Yes | 6.781307 | 1.05E-05 | 4.53E-04 |
| *Lactobacillus_saerimneri* | MHE_on_PHES_Yes | 5.035906 | 1.26E-05 | 5.23E-04 |
| *Bacteroides_coprocola* | MHE_on_PHES_No | 2.935415 | 1.39E-05 | 5.59E-04 |
| *Lactobacillus_gallinarum* | MHE_on_PHES_Yes | 5.030438 | 1.67E-05 | 6.52E-04 |
| *Pantoea_coffeiphila* | MHE_on_PHES_Yes | 6.209209 | 2.11E-05 | 7.95E-04 |
| *Clostridium_sp._Marseille_P2415* | MHE_on_PHES_No | 5.907781 | 2.27E-05 | 8.28E-04 |
| *Klebsiella_sp._MBT_K_1* | MHE_on_PHES_Yes | 5.49493 | 2.83E-05 | 0.001004 |
| *Megamonas_hypermegale* | MHE_on_PHES_Yes | 7.143223 | 3.02E-05 | 0.00104 |
| *Enterococcus_faecalis* | MHE_on_PHES_No | 3.902145 | 3.15E-05 | 0.001056 |
| *Veillonella_seminalis* | MHE_on_PHES_No | 5.415206 | 4.18E-05 | 0.001332 |
| *Serratia_sp._YD25* | MHE_on_PHES_Yes | 5.80664 | 4.20E-05 | 0.001332 |
| *Proteus_columbae* | MHE_on_PHES_Yes | 6.292186 | 5.17E-05 | 0.0016 |
| *Lactobacillus_kalixensis* | MHE_on_PHES_Yes | 4.659796 | 5.48E-05 | 0.001651 |
| *Lactobacillus_parakefiri* | MHE_on_PHES_Yes | 4.567468 | 6.42E-05 | 0.001889 |
| *Fusobacterium_varium* | MHE_on_PHES_Yes | 6.161832 | 7.01E-05 | 0.001991 |
| *Neisseria_sp._GT4A_CT1* | MHE_on_PHES_Yes | 6.045774 | 7.10E-05 | 0.001991 |
| *Fusobacterium_sp._HMSC073F01* | MHE_on_PHES_Yes | 5.918339 | 8.00E-05 | 0.002191 |
| *Faecalicatena_fissicatena* | MHE_on_PHES_Yes | 4.2332 | 8.18E-05 | 0.002191 |
| *Prevotella_oralis* | MHE_on_PHES_Yes | 6.599951 | 8.79E-05 | 0.002304 |
| *Dakarella_massiliensis* | MHE_on_PHES_No | 3.690501 | 9.24E-05 | 0.00237 |
| *Bifidobacterium_crudilactis* | MHE_on_PHES_Yes | 6.300786 | 1.08E-04 | 0.002674 |
| *Prevotella_maculosa* | MHE_on_PHES_Yes | 4.79873 | 1.09E-04 | 0.002674 |
| *Klebsiella_pneumoniae* | MHE_on_PHES_Yes | 2.349655 | 1.11E-04 | 0.002682 |
| *Prevotella_saccharolytica* | MHE_on_PHES_Yes | 4.821261 | 1.15E-04 | 0.002731 |
| *Cuspidothrix_issatschenkoi* | MHE_on_PHES_Yes | 5.154216 | 1.18E-04 | 0.002746 |
| *Tetzosporium_hominis* | MHE_on_PHES_Yes | 5.594711 | 1.23E-04 | 0.002792 |
| *Paenibacillus_sp._VT_16_81* | MHE_on_PHES_Yes | 4.825007 | 1.34E-04 | 0.00299 |
| **Lactulose use** | MHE_on_PHES_Yes | 0.693147 | 1.38E-04 | 0.00299 |
| *Yersinia_frederiksenii* | MHE_on_PHES_Yes | 5.136194 | 1.39E-04 | 0.00299 |
| *Lactobacillus_ingluviei* | MHE_on_PHES_Yes | 4.707526 | 1.46E-04 | 0.003088 |
| *Megasphaera_micronuciformis* | MHE_on_PHES_Yes | 2.262242 | 1.53E-04 | 0.003177 |
| *Proteus_sp._HMSC10D02* | MHE_on_PHES_Yes | 4.772602 | 1.57E-04 | 0.003206 |
| *Butyrivibrio_crossotus* | MHE_on_PHES_No | 3.212415 | 1.82E-04 | 0.003656 |
| *Caecibacter_massiliensis* | MHE_on_PHES_Yes | 3.422903 | 1.93E-04 | 0.003818 |
| *Lactobacillus_curvatus* | MHE_on_PHES_No | 2.8572 | 2.10E-04 | 0.004092 |
| *Lactobacillus_pontis* | MHE_on_PHES_Yes | 5.279274 | 2.23E-04 | 0.004275 |
| *Clostridiales_bacterium_CHKCI001* | MHE_on_PHES_No | 1.791372 | 2.28E-04 | 0.004293 |
| *Prevotella_nigrescens* | MHE_on_PHES_Yes | 4.344796 | 2.32E-04 | 0.004305 |
| *Bifidobacterium_kashiwanohense* | MHE_on_PHES_No | 2.474408 | 2.57E-04 | 0.004704 |
| *Pseudoramibacter_alactolyticus* | MHE_on_PHES_Yes | 4.250799 | 2.64E-04 | 0.004754 |
| *Anaeroglobus_geminatus* | MHE_on_PHES_Yes | 4.264439 | 2.78E-04 | 0.00492 |
| *Turicibacter_sp._H121* | MHE_on_PHES_Yes | 6.531849 | 2.82E-04 | 0.00492 |
| *Alcanivorax_hongdengensis* | MHE_on_PHES_Yes | 5.079247 | 2.95E-04 | 0.005077 |
| *Bifidobacterium_breve* | MHE_on_PHES_Yes | 1.831566 | 3.05E-04 | 0.005137 |
| *Lactococcus_sp._DD01* | MHE_on_PHES_Yes | 4.713656 | 3.07E-04 | 0.005137 |
| *Porphyromonas_sp._KLE_1280* | MHE_on_PHES_No | 2.639057 | 3.22E-04 | 0.005149 |
| *Streptococcus_sp._HMSC072D07* | MHE_on_PHES_Yes | 2.97205 | 3.31E-04 | 0.005149 |
| *Leptospira_sp._JW3_C_A1* | MHE_on_PHES_Yes | 4.893507 | 3.37E-04 | 0.005149 |
| *Staphylococcus_fleurettii* | MHE_on_PHES_Yes | 3.987559 | 3.49E-04 | 0.005149 |
| *Victivallales_bacterium_CCUG_44730* | MHE_on_PHES_Yes | 3.315112 | 3.49E-04 | 0.005149 |
| *Prevotella_sp._oral_taxon_299* | MHE_on_PHES_Yes | 4.775141 | 3.51E-04 | 0.005149 |
| *Paeniglutamicibacter_antarcticus* | MHE_on_PHES_Yes | 4.916779 | 3.54E-04 | 0.005149 |
| ***Prior HE*** | MHE_on_PHES_Yes | 0.559616 | 3.54E-04 | 0.005149 |
| *Streptococcus_sp._HMSC076C08* | MHE_on_PHES_Yes | 2.829678 | 3.55E-04 | 0.005149 |
| *Prevotella_sp._HMSC069G02* | MHE_on_PHES_Yes | 2.932011 | 3.56E-04 | 0.005149 |
| *Bifidobacterium_sp._AGR2158* | MHE_on_PHES_Yes | 3.753418 | 3.59E-04 | 0.005149 |
| *Siccibacter_colletis* | MHE_on_PHES_Yes | 5.097686 | 3.62E-04 | 0.005149 |
| ***Rifaximin use*** | MHE_on_PHES_Yes | 0.961411 | 3.64E-04 | 0.005149 |
| *Leuconostoc_pseudomesenteroides* | MHE_on_PHES_Yes | 4.21817 | 3.67E-04 | 0.005149 |
| *Prevotella_denticola* | MHE_on_PHES_Yes | 2.753107 | 3.84E-04 | 0.005267 |
| *Bifidobacterium_gallinarum* | MHE_on_PHES_Yes | 4.135871 | 3.90E-04 | 0.005267 |
| *Aerococcus_sp._HMSC23C02* | MHE_on_PHES_Yes | 4.857108 | 3.93E-04 | 0.005267 |
| *Duodenibacillus_massiliensis* | MHE_on_PHES_No | 3.153175 | 3.93E-04 | 0.005267 |
| *Lactobacillus_amylolyticus* | MHE_on_PHES_Yes | 13.01778 | 4.07E-04 | 0.005391 |
| *Prevotella_marshii* | MHE_on_PHES_Yes | 2.764086 | 4.13E-04 | 0.005396 |
| *Prevotella_sp._HMSC073D09* | MHE_on_PHES_Yes | 4.765742 | 4.16E-04 | 0.005396 |
| *Propionibacterium_sp._HMSC067A02* | MHE_on_PHES_Yes | 4.868816 | 4.71E-04 | 0.006045 |
| *Kluyvera_cryocrescens* | MHE_on_PHES_Yes | 4.368405 | 5.00E-04 | 0.006344 |
| *Bifidobacterium_sp._TRE_1* | MHE_on_PHES_Yes | 2.723173 | 5.36E-04 | 0.006662 |
| **Age** | MHE_on_PHES_Yes | 0.092148 | 5.37E-04 | 0.006662 |
| *Blautia_producta* | MHE_on_PHES_No | 2.913157 | 5.41E-04 | 0.006662 |
| *Lactobacillus_sp._HMSC073D04* | MHE_on_PHES_Yes | 3.881564 | 5.60E-04 | 0.006824 |
| *Porphyromonas_gingivalis* | MHE_on_PHES_Yes | 5.022085 | 5.72E-04 | 0.006899 |
| *Streptococcus_sp._HMSC034B05* | MHE_on_PHES_No | 1.789781 | 6.19E-04 | 0.007388 |
| *Colwellia_echini* | MHE_on_PHES_Yes | 4.617383 | 6.31E-04 | 0.007427 |
| *Collinsella_stercoris* | MHE_on_PHES_No | 2.852301 | 6.34E-04 | 0.007427 |
| *Obesumbacterium_proteus* | MHE_on_PHES_No | 4.787223 | 6.49E-04 | 0.007529 |
| *Lactobacillus_rossiae* | MHE_on_PHES_Yes | 4.545823 | 6.72E-04 | 0.007646 |
| *Lactobacillus_sp._HMSC073B09* | MHE_on_PHES_Yes | 4.144268 | 6.81E-04 | 0.007646 |
| *Serratia_sp._Leaf51* | MHE_on_PHES_Yes | 5.613128 | 6.88E-04 | 0.007646 |
| *Selenomonas_sputigena* | MHE_on_PHES_Yes | 1.914673 | 6.93E-04 | 0.007646 |
| *Pediococcus_acidilactici* | MHE_on_PHES_No | 4.106653 | 7.03E-04 | 0.007646 |
| *Prevotella_albensis* | MHE_on_PHES_No | 1.672906 | 7.06E-04 | 0.007646 |
| *Bacillus_paralicheniformis* | MHE_on_PHES_Yes | 4.717044 | 7.09E-04 | 0.007646 |
| *Yersinia_enterocolitica* | MHE_on_PHES_No | 4.331869 | 7.10E-04 | 0.007646 |
| *Citrobacter_freundii_complex_sp._CFNIH2* | MHE_on_PHES_Yes | 4.409003 | 8.29E-04 | 0.008847 |
| *Erysipelothrix_larvae* | MHE_on_PHES_Yes | 6.019404 | 8.64E-04 | 0.009139 |
| *Klebsiella_sp._MS_92_3* | MHE_on_PHES_Yes | 2.543153 | 9.09E-04 | 0.009528 |
| *Enterococcus_sp._HMSC14A10* | MHE_on_PHES_Yes | 4.179502 | 9.89E-04 | 0.010198 |
| *Coprobacillus_sp._29_1* | MHE_on_PHES_Yes | 1.512888 | 9.89E-04 | 0.010198 |
| *Lactobacillus_pasteurii* | MHE_on_PHES_No | 4.436751 | 0.001023 | 0.010455 |
| *Neisseria_gonorrhoeae* | MHE_on_PHES_Yes | 4.429468 | 0.001081 | 0.010954 |
| *Citrobacter_sp._MGH100* | MHE_on_PHES_Yes | 2.877885 | 0.001103 | 0.011086 |
| *Acidaminococcus_fermentans* | MHE_on_PHES_Yes | 3.383106 | 0.001136 | 0.011323 |
| *Lactobacillus_gasseri* | MHE_on_PHES_No | 1.911808 | 0.001225 | 0.012107 |
| *Clostridium_sp._KLE_1755* | MHE_on_PHES_No | 2.700401 | 0.001242 | 0.012181 |
| *Streptococcus_sp._HMSC071H03* | MHE_on_PHES_No | 3.291909 | 0.001445 | 0.014052 |
| *Lachnospiraceae_bacterium_KHCPX20* | MHE_on_PHES_No | 2.045994 | 0.001564 | 0.01509 |
| *Tannerella_forsythia* | MHE_on_PHES_Yes | 4.420947 | 0.001591 | 0.015227 |
| *Enterobacter_cloacae_complex_bacterium* | MHE_on_PHES_No | 4.413406 | 0.001704 | 0.016182 |
| *Lactobacillus_salivarius* | MHE_on_PHES_No | 3.199219 | 0.001733 | 0.01633 |
| *Lachnospiraceae_bacterium_2_1_46FAA* | MHE_on_PHES_Yes | 1.564709 | 0.001856 | 0.017255 |
| *Bacteroides_sp._4_3_47FAA* | MHE_on_PHES_Yes | 1.439559 | 0.00186 | 0.017255 |
| *Prevotella_jejuni* | MHE_on_PHES_Yes | 3.941128 | 0.001916 | 0.017623 |
| *Pediococcus_inopinatus* | MHE_on_PHES_No | 4.625605 | 0.001929 | 0.017623 |
| *Akkermansia_sp._KLE1797* | MHE_on_PHES_No | 3.085936 | 0.001955 | 0.017729 |
| *Porphyromonas_gulae* | MHE_on_PHES_No | 3.347698 | 0.00209 | 0.01881 |
| *Fusobacterium_nucleatum* | MHE_on_PHES_Yes | 3.049237 | 0.002141 | 0.019026 |
| *bacterium_OL_1* | MHE_on_PHES_Yes | 3.373958 | 0.002146 | 0.019026 |
| *Anaerotruncus_sp._AT3* | MHE_on_PHES_Yes | 3.359364 | 0.00226 | 0.019759 |
| *Prevotella_disiens* | MHE_on_PHES_No | 1.871225 | 0.002264 | 0.019759 |
| *Clostridium_hylemonae* | MHE_on_PHES_No | 2.360842 | 0.002277 | 0.019759 |
| *Dialister_invisus* | MHE_on_PHES_No | 1.534848 | 0.002406 | 0.020726 |
| *Prevotella_lascolaii* | MHE_on_PHES_No | 1.45801 | 0.002443 | 0.020899 |
| *Urmitella_timonensis* | MHE_on_PHES_No | 2.487074 | 0.002481 | 0.021074 |
| *Cronobacter_malonaticus* | MHE_on_PHES_Yes | 3.500932 | 0.002589 | 0.021837 |
| *Clostridium_disporicum* | MHE_on_PHES_Yes | 3.431926 | 0.002632 | 0.021931 |
| *Lactobacillus_johnsonii* | MHE_on_PHES_Yes | 3.121116 | 0.002637 | 0.021931 |
| *Alistipes_ihumii* | MHE_on_PHES_No | 1.714438 | 0.002785 | 0.023004 |
| *Lactobacillus_rhamnosus* | MHE_on_PHES_Yes | 2.714804 | 0.002882 | 0.023643 |
| *Dysgonomonas_gadei* | MHE_on_PHES_Yes | 4.163061 | 0.002977 | 0.024256 |
| *Megasphaera_cerevisiae* | MHE_on_PHES_Yes | 3.566531 | 0.003059 | 0.024756 |
| *Klebsiella_quasivariicola* | MHE_on_PHES_Yes | 1.795405 | 0.003275 | 0.026332 |
| *Citrobacter_sp._30_2* | MHE_on_PHES_Yes | 3.142587 | 0.003328 | 0.026438 |
| *Lactobacillus_sp._FMNP02* | MHE_on_PHES_Yes | 3.477683 | 0.003332 | 0.026438 |
| *Fusobacterium_ulcerans* | MHE_on_PHES_Yes | 3.548003 | 0.003598 | 0.028358 |
| *Bifidobacterium_callitrichos* | MHE_on_PHES_Yes | 2.334222 | 0.003643 | 0.028532 |
| *Klebsiella_oxytoca* | MHE_on_PHES_No | 3.002784 | 0.004017 | 0.031254 |
| *Actinomyces_radicidentis* | MHE_on_PHES_No | 3.913612 | 0.004161 | 0.032124 |
| *Lactobacillus_agilis* | MHE_on_PHES_No | 2.954683 | 0.004182 | 0.032124 |
| *Fastidiosipila_sanguinis* | MHE_on_PHES_No | 1.953128 | 0.004339 | 0.033122 |
| *Prevotella_loescheii* | MHE_on_PHES_Yes | 3.833743 | 0.004655 | 0.03531 |
| *Cellulomonas_carbonis* | MHE_on_PHES_No | 1.531055 | 0.004839 | 0.036337 |
| *Bacteroides_cellulosilyticus* | MHE_on_PHES_No | 1.636428 | 0.004863 | 0.036337 |
| *Klebsiella_sp._X1_16S_Nf21* | MHE_on_PHES_Yes | 1.538478 | 0.004881 | 0.036337 |
| *Gardnerella_sp._30_4* | MHE_on_PHES_Yes | 3.684181 | 0.00493 | 0.036399 |
| *Klebsiella_sp._AA405* | MHE_on_PHES_Yes | 2.233744 | 0.00495 | 0.036399 |
| *Klebsiella_sp._1_1_55* | MHE_on_PHES_Yes | 2.662738 | 0.00508 | 0.037129 |
| *Streptococcus_sp._HMSC067A03* | MHE_on_PHES_Yes | 2.867899 | 0.005118 | 0.037182 |
| *Streptococcus_sp._A12* | MHE_on_PHES_No | 1.465971 | 0.005244 | 0.037871 |
| *Streptococcus_australis* | MHE_on_PHES_No | 1.241357 | 0.005347 | 0.038384 |
| *Alcohol_etiology* | MHE_on_PHES_Yes | 0.693147 | 0.005422 | 0.038637 |
| *Akkermansia_sp._KLE1798* | MHE_on_PHES_No | 3.003028 | 0.005446 | 0.038637 |
| *Rikenella_microfusus* | MHE_on_PHES_No | 1.570452 | 0.005531 | 0.039011 |
| **Gender** | MHE_on_PHES_Yes | 0.287682 | 0.005599 | 0.039255 |
| Citrobacter_werkmanii | MHE_on_PHES_Yes | 2.000261 | 0.005844 | 0.040521 |
| Emergencia_timonensis | MHE_on_PHES_No | 1.499376 | 0.00586 | 0.040521 |
| Klebsiella_sp._LTGPAF_6F | MHE_on_PHES_Yes | 2.995732 | 0.00588 | 0.040521 |
| Lactobacillus_pentosus | MHE_on_PHES_No | 3.282277 | 0.005938 | 0.040688 |
| Leptotrichia_sp._oral_taxon_215 | MHE_on_PHES_No | 2.290006 | 0.006325 | 0.043094 |
| Klebsiella_sp._HMSC16A12 | MHE_on_PHES_Yes | 2.174458 | 0.006617 | 0.044782 |
| Clostridium_saccharolyticum | MHE_on_PHES_No | 0.786289 | 0.006647 | 0.044782 |
| Olsenella_scatoligenes | MHE_on_PHES_Yes | 2.195244 | 0.006935 | 0.046467 |
| Sutterella_wadsworthensis | MHE_on_PHES_Yes | 1.447871 | 0.007218 | 0.047915 |
| Akkermansia_sp._KLE1605 | MHE_on_PHES_No | 3.037752 | 0.007231 | 0.047915 |
| Clostridium_saccharogumia | MHE_on_PHES_Yes | 1.394515 | 0.00742 | 0.048898 |
| Romboutsia_timonensis | MHE_on_PHES_No | 1.581238 | 0.007482 | 0.049038 |

**Table S2: MAASlin2 analysis of all MHE-PHES versus not bacterial species**

| **Feature** | **Higher in** | **Coefficient** | **P-Value** | **Q-Value** |
| --- | --- | --- | --- | --- |
| *Clostridium_aerotolerans* | MHE_on_ICT_No | 8.528026 | 6.66E-18 | 8.04E-15 |
| *Ruminococcaceae_bacterium_CPB6* | MHE_on_ICT_No | 8.150279 | 2.92E-16 | 1.76E-13 |
| *Enterococcus_sp._HMSC064A12* | MHE_on_ICT_No | 7.352023 | 8.57E-16 | 3.45E-13 |
| *Enterococcus_sp._HMSC035B04* | MHE_on_ICT_No | 6.790699 | 1.09E-14 | 3.29E-12 |
| *Enterococcus_faecium* | MHE_on_ICT_No | 4.997246 | 1.43E-14 | 3.46E-12 |
| *Enterococcus_sp._HMSC076D08* | MHE_on_ICT_No | 6.869707 | 5.82E-14 | 1.17E-11 |
| *Clostridium_sp._Marseille_P2415* | MHE_on_ICT_No | 6.134822 | 1.49E-12 | 2.57E-10 |
| *Enterococcus_sp._HMSC070F12* | MHE_on_ICT_No | 6.983724 | 7.14E-12 | 1.08E-09 |
| *Veillonella_seminalis* | MHE_on_ICT_No | 6.416805 | 1.06E-10 | 1.35E-08 |
| *Enterococcus_sp._HMSC077E04* | MHE_on_ICT_No | 5.913945 | 1.12E-10 | 1.35E-08 |
| *Enterococcus_sp._HMSC073E08* | MHE_on_ICT_No | 6.045294 | 2.71E-10 | 2.98E-08 |
| *Ethanoligenens_harbinense* | MHE_on_ICT_No | 6.423644 | 7.23E-10 | 7.27E-08 |
| *Lactobacillus_mucosae* | MHE_on_ICT_No | 5.034449 | 1.50E-09 | 1.39E-07 |
| *Enterococcus_sp._HMSC065H03* | MHE_on_ICT_No | 5.679734 | 2.68E-09 | 2.31E-07 |
| *Enterococcus_sp._HMSC077E07* | MHE_on_ICT_No | 6.492352 | 3.67E-09 | 2.95E-07 |
| *Enterococcus_sp._HMSC060E05* | MHE_on_ICT_No | 5.67358 | 9.69E-09 | 7.31E-07 |
| *Enterococcus_sp._HMSC060D09* | MHE_on_ICT_No | 6.245553 | 1.09E-08 | 7.72E-07 |
| *Enterococcus_gallinarum* | MHE_on_ICT_No | 3.842571 | 1.98E-08 | 1.33E-06 |
| *Dysgonomonas_capnocytophagoides* | MHE_on_ICT_No | 4.257087 | 5.64E-08 | 3.58E-06 |
| *Pseudopropionibacterium_propionicum* | MHE_on_ICT_No | 4.569408 | 6.24E-08 | 3.77E-06 |
| *Pseudomonas_stutzeri* | MHE_on_ICT_No | 11.07628 | 6.59E-08 | 3.79E-06 |
| *Enterococcus_sp._HMSC056C08* | MHE_on_ICT_No | 5.558158 | 7.27E-08 | 3.99E-06 |
| *Parvimonas_micra* | MHE_on_ICT_No | 3.814043 | 1.08E-07 | 5.65E-06 |
| *Enterococcus_sp._HMSC034B11* | MHE_on_ICT_No | 4.949215 | 2.86E-07 | 1.40E-05 |
| *Atopobium_minutum* | MHE_on_ICT_No | 5.735377 | 2.91E-07 | 1.40E-05 |
| *Desulfovibrio_sp._6_1_46AFAA* | MHE_on_ICT_No | 6.271971 | 5.54E-07 | 2.57E-05 |
| *Lactobacillus_sakei* | MHE_on_ICT_No | 3.698148 | 6.35E-07 | 2.84E-05 |
| *Dakarella_massiliensis* | MHE_on_ICT_Yes | 6.124379 | 6.98E-07 | 3.01E-05 |
| *Turicibacter_sp._H121* | MHE_on_ICT_No | 5.375149 | 7.76E-07 | 3.23E-05 |
| *Clostridium_hylemonae* | MHE_on_ICT_No | 3.270158 | 1.62E-06 | 6.51E-05 |
| *Enterococcus_asini* | MHE_on_ICT_No | 3.722906 | 3.17E-06 | 1.23E-04 |
| *Enterococcus_sp._HMSC14A10* | MHE_on_ICT_No | 4.283045 | 3.40E-06 | 1.28E-04 |
| *Desulfovibrio_fairfieldensis* | MHE_on_ICT_No | 6.235278 | 3.58E-06 | 1.31E-04 |
| *Slackia_exigua* | MHE_on_ICT_No | 3.392962 | 4.81E-06 | 1.71E-04 |
| *Megasphaera_genomosp._type_2* | MHE_on_ICT_No | 3.399195 | 9.05E-06 | 3.12E-04 |
| *Olsenella_umbonata* | MHE_on_ICT_No | 3.229962 | 1.05E-05 | 3.52E-04 |
| *Enterococcus_sp._HMSC072D11* | MHE_on_ICT_No | 4.96136 | 1.80E-05 | 5.86E-04 |
| *Citrobacter_freundii_complex_sp._CFNIH2* | MHE_on_ICT_No | 3.544912 | 1.90E-05 | 6.03E-04 |
| *Collinsella_ihuae* | MHE_on_ICT_No | 3.088538 | 2.01E-05 | 6.22E-04 |
| *Enterococcus_sp._HMSC076E04* | MHE_on_ICT_No | 3.802299 | 3.45E-05 | 0.001041 |
| *Actinomyces_sp._oral_taxon_897* | MHE_on_ICT_No | 3.845597 | 4.04E-05 | 0.001189 |
| *Prevotella_sp._Marseille_P4119* | MHE_on_ICT_No | 2.547023 | 4.49E-05 | 0.001291 |
| *Clostridium_sp._ASF502* | MHE_on_ICT_No | 1.720009 | 6.98E-05 | 0.00196 |
| *Enterococcus_sp._HMSC035C10* | MHE_on_ICT_No | 3.996174 | 9.04E-05 | 0.00248 |
| *Clostridium_bolteae* | MHE_on_ICT_No | 2.430375 | 9.53E-05 | 0.002531 |
| *Erysipelotrichaceae_bacterium_2_2_44A* | MHE_on_ICT_No | 0.89349 | 9.65E-05 | 0.002531 |
| *Enterococcus_sp._HMSC063D12* | MHE_on_ICT_No | 3.32556 | 1.00E-04 | 0.002577 |
| *Lactobacillus_pasteurii* | MHE_on_ICT_No | 3.343078 | 1.13E-04 | 0.00284 |
| *Enterococcus_sp._HMSC063H10* | MHE_on_ICT_No | 3.815932 | 1.44E-04 | 0.00355 |
| *Blautia_producta* | MHE_on_ICT_No | 3.298523 | 1.63E-04 | 0.003927 |
| *Actinomyces_israelii* | MHE_on_ICT_No | 3.076187 | 2.52E-04 | 0.005869 |
| *Shigella_dysenteriae* | MHE_on_ICT_Yes | 2.163442 | 2.53E-04 | 0.005869 |
| *Shigella_boydii* | MHE_on_ICT_Yes | 2.180324 | 2.58E-04 | 0.005873 |
| *Megasphaera_massiliensis* | MHE_on_ICT_Yes | 3.215087 | 3.40E-04 | 0.007588 |
| *Cloacibacillus_porcorum* | MHE_on_ICT_Yes | 5.59103 | 6.10E-04 | 0.013238 |
| *Bifidobacterium_minimum* | MHE_on_ICT_Yes | 3.406066 | 6.14E-04 | 0.013238 |
| *Desulfotomaculum_guttoideum* | MHE_on_ICT_No | 2.806037 | 7.34E-04 | 0.015539 |
| *Collinsella_sp._Marseille_P3740* | MHE_on_ICT_No | 1.864922 | 7.49E-04 | 0.015581 |
| *Absiella_dolichum* | MHE_on_ICT_No | 1.718104 | 7.65E-04 | 0.015648 |
| *Shigella_flexneri* | MHE_on_ICT_Yes | 2.158204 | 7.95E-04 | 0.016003 |
| *Gemella_morbillorum* | MHE_on_ICT_No | 1.594121 | 8.96E-04 | 0.017729 |
| *Bifidobacterium_kashiwanohense* | MHE_on_ICT_No | 2.943553 | 9.36E-04 | 0.017995 |
| *Eubacterium_nodatum* | MHE_on_ICT_No | 2.104839 | 9.48E-04 | 0.017995 |
| *Prevotella_copri* | MHE_on_ICT_Yes | 2.20677 | 9.56E-04 | 0.017995 |
| *Enterococcus_sp._HMSC072F02* | MHE_on_ICT_No | 3.494618 | 9.69E-04 | 0.017995 |
| *Ruminococcus_callidus* | MHE_on_ICT_Yes | 3.179383 | 0.001066 | 0.019492 |
| *Enterococcus_sp._3G1_DIV0629* | MHE_on_ICT_No | 3.575152 | 0.001127 | 0.020299 |
| *Fusobacterium_sp._HMSC073F01* | MHE_on_ICT_Yes | 16.38437 | 0.001183 | 0.021007 |
| *Roseburia_intestinalis* | MHE_on_ICT_No | 2.062827 | 0.001216 | 0.021273 |
| *Desulfovibrio_legallii* | MHE_on_ICT_Yes | 4.902546 | 0.001296 | 0.022355 |
| *Lactococcus_piscium* | MHE_on_ICT_No | 2.962224 | 0.00136 | 0.023096 |
| *Clostridium_sp._Marseille_P3244* | MHE_on_ICT_No | 1.643515 | 0.001395 | 0.023096 |
| *Fusobacterium_ulcerans* | MHE_on_ICT_Yes | 6.265139 | 0.001397 | 0.023096 |
| *Murdochiella_vaginalis* | MHE_on_ICT_No | 1.026069 | 0.001437 | 0.023438 |
| *Enterococcus_sp._HMSC067C01* | MHE_on_ICT_No | 2.646468 | 0.001527 | 0.024572 |
| *Leuconostoc_citreum* | MHE_on_ICT_No | 3.085084 | 0.001733 | 0.02752 |
| *Bacteroides_sp._2_1_33B* | MHE_on_ICT_No | 1.324784 | 0.002081 | 0.032621 |
| *Providencia_rettgeri* | MHE_on_ICT_Yes | 14.8688 | 0.002129 | 0.032943 |
| *Ruminococcus_bromii* | MHE_on_ICT_No | 1.62031 | 0.002512 | 0.038213 |
| *Peptostreptococcus_stomatis* | MHE_on_ICT_No | 2.079511 | 0.002533 | 0.038213 |
| *Enterococcus_sp._5B3_DIV0040* | MHE_on_ICT_No | 3.249762 | 0.00258 | 0.03844 |
| *Lachnospiraceae_bacterium_3_1* | MHE_on_ICT_No | 0.847338 | 0.002887 | 0.042496 |
| *Lactobacillus_harbinensis* | MHE_on_ICT_Yes | 6.89784 | 0.002973 | 0.04323 |
| *Veillonella_parvula* | MHE_on_ICT_Yes | 2.01719 | 0.003309 | 0.047541 |

**Table S3: MAASlin2 analysis of** **bacterial species in discordant patients**

| **Feature** | **Higher in** | **Coefficient** | **P-Value** | **Q-Value** |
| --- | --- | --- | --- | --- |
| *Turicibacter_sp._H121* | PHES_1_ICT_0 | 16.05104 | 1.25E-16 | 1.50E-13 |
| *Enterococcus_sp._HMSC064A12* | PHES_1_ICT_0 | 8.103585 | 1.29E-13 | 7.75E-11 |
| *Enterococcus_sp._HMSC035B04* | PHES_1_ICT_0 | 7.726573 | 2.61E-13 | 1.05E-10 |
| *Enterococcus_sp._HMSC14A10* | PHES_1_ICT_0 | 7.102974 | 1.58E-11 | 4.76E-09 |
| *Citrobacter_freundii_complex_sp._CFNIH2* | PHES_1_ICT_0 | 6.902109 | 1.53E-10 | 3.69E-08 |
| *Enterococcus_sp._HMSC073E08* | PHES_1_ICT_0 | 7.416716 | 7.24E-10 | 1.45E-07 |
| *Proteus_mirabilis* | PHES_1_ICT_0 | 7.246815 | 2.31E-09 | 3.51E-07 |
| *Enterococcus_sp._HMSC065H03* | PHES_1_ICT_0 | 6.86829 | 2.33E-09 | 3.51E-07 |
| *Enterococcus_sp._HMSC076D08* | PHES_1_ICT_0 | 7.728452 | 2.83E-09 | 3.79E-07 |
| *Enterococcus_sp._HMSC070F12* | PHES_1_ICT_0 | 7.624099 | 3.42E-09 | 4.12E-07 |
| *Enterococcus_sp._HMSC077E07* | PHES_1_ICT_0 | 7.649075 | 4.00E-09 | 4.38E-07 |
| *Enterococcus_sp._HMSC060E05* | PHES_1_ICT_0 | 6.297979 | 1.27E-08 | 1.27E-06 |
| *Clostridium_sp._BNL1100* | PHES_1_ICT_0 | 11.38166 | 2.54E-08 | 2.31E-06 |
| *Enterococcus_sp._HMSC056C08* | PHES_1_ICT_0 | 6.738126 | 2.69E-08 | 2.31E-06 |
| *Enterococcus_sp._HMSC034B11* | PHES_1_ICT_0 | 6.198366 | 4.17E-08 | 3.34E-06 |
| *Enterococcus_gallinarum* | PHES_1_ICT_0 | 4.599268 | 1.34E-07 | 1.01E-05 |
| *Enterococcus_sp._HMSC077E04* | PHES_1_ICT_0 | 6.860062 | 2.41E-07 | 1.71E-05 |
| *Enterococcus_asini* | PHES_1_ICT_0 | 4.573156 | 5.91E-07 | 3.95E-05 |
| *Streptococcus_sp._HMSC072D07* | PHES_1_ICT_0 | 4.370385 | 6.32E-07 | 4.00E-05 |
| *Eisenbergiella_tayi* | PHES_1_ICT_0 | 3.482444 | 1.56E-06 | 9.38E-05 |
| *Prevotella_copri* | PHES_0_ICT_1 | 5.29871 | 1.64E-06 | 9.40E-05 |
| *Clostridium_sp._ASF502* | PHES_1_ICT_0 | 2.269838 | 1.73E-06 | 9.48E-05 |
| *Streptococcus_sp._HMSC076C08* | PHES_1_ICT_0 | 3.799601 | 1.23E-05 | 6.44E-04 |
| *Dysgonomonas_capnocytophagoides* | PHES_1_ICT_0 | 5.796443 | 1.67E-05 | 8.35E-04 |
| *Prevotella_sp._Marseille_P4119* | PHES_1_ICT_0 | 3.327505 | 2.27E-05 | 0.001094 |
| *Enterococcus_sp._HMSC072F02* | PHES_1_ICT_0 | 5.033981 | 2.73E-05 | 0.001262 |
| *Megasphaera_massiliensis* | PHES_0_ICT_1 | 6.105243 | 3.14E-05 | 0.001399 |
| *Eggerthella_sp._YY7918* | PHES_0_ICT_1 | 4.905184 | 3.58E-05 | 0.001539 |
| *Alistipes_ihumii* | PHES_0_ICT_1 | 5.106802 | 3.83E-05 | 0.001589 |
| *Prevotella_oralis* | PHES_1_ICT_0 | 5.365457 | 4.50E-05 | 0.001804 |
| *Enterococcus_sp._HMSC072D11* | PHES_1_ICT_0 | 5.860311 | 5.36E-05 | 0.002073 |
| *Roseburia_intestinalis* | PHES_1_ICT_0 | 3.575024 | 5.59E-05 | 0.002073 |
| *Clostridium_sp._D5* | PHES_1_ICT_0 | 1.953824 | 5.69E-05 | 0.002073 |
| *Bifidobacterium_adolescentis* | PHES_0_ICT_1 | 4.318512 | 8.42E-05 | 0.002912 |
| *Pseudoflavonifractor_capillosus* | PHES_1_ICT_0 | 2.814878 | 8.47E-05 | 0.002912 |
| *Citrobacter_werkmanii* | PHES_1_ICT_0 | 3.236842 | 1.21E-04 | 0.004039 |
| *Romboutsia_timonensis* | PHES_0_ICT_1 | 6.641835 | 1.33E-04 | 0.004327 |
| *Leuconostoc_citreum* | PHES_1_ICT_0 | 4.975464 | 1.55E-04 | 0.004921 |
| *Dakarella_massiliensis* | PHES_0_ICT_1 | 6.439336 | 1.63E-04 | 0.005032 |
| *Enterococcus_sp._HMSC076E04* | PHES_1_ICT_0 | 5.226286 | 1.68E-04 | 0.005049 |
| *Varibaculum_cambriense* | PHES_1_ICT_0 | 2.622651 | 1.89E-04 | 0.005523 |
| *Selenomonas_bovis* | PHES_1_ICT_0 | 5.347108 | 1.93E-04 | 0.005523 |
| *Eggerthellaceae_bacterium_AT8* | PHES_1_ICT_0 | 4.982464 | 2.35E-04 | 0.006573 |
| *Enterococcus_sp._3G1_DIV0629* | PHES_1_ICT_0 | 5.184204 | 2.42E-04 | 0.006617 |
| *Enterococcus_sp._HMSC063D12* | PHES_1_ICT_0 | 5.003774 | 2.95E-04 | 0.007881 |
| *Lactococcus_raffinolactis* | PHES_1_ICT_0 | 3.371049 | 5.35E-04 | 0.014004 |
| *Serratia_sp._YD25* | PHES_1_ICT_0 | 4.471639 | 5.49E-04 | 0.014039 |
| *Eubacterium_sp._SB2* | PHES_0_ICT_1 | 4.540933 | 5.87E-04 | 0.014724 |
| *Bifidobacterium_mongoliense* | PHES_1_ICT_0 | 4.70048 | 7.33E-04 | 0.017992 |
| *Clostridium_perfringens* | PHES_1_ICT_0 | 3.10689 | 8.33E-04 | 0.019956 |
| *Chryseobacterium_bovis* | PHES_1_ICT_0 | 3.577213 | 8.46E-04 | 0.019956 |
| *Streptococcus_infantarius* | PHES_1_ICT_0 | 2.701288 | 8.90E-04 | 0.020591 |
| *Yersinia_kristensenii* | PHES_1_ICT_0 | 3.601868 | 0.00115 | 0.0261 |
| *Absiella_dolichum* | PHES_1_ICT_0 | 2.325958 | 0.001256 | 0.02799 |
| *Bacteroides_fluxus* | PHES_0_ICT_1 | 2.762113 | 0.001482 | 0.032421 |
| *Streptococcus_sp._HMSC067A03* | PHES_1_ICT_0 | 3.02675 | 0.001731 | 0.037184 |
| *Lactobacillus_zeae* | PHES_1_ICT_0 | 2.998333 | 0.001859 | 0.039239 |
| *Streptococcus_sp._HPH0090* | PHES_1_ICT_0 | 2.502835 | 0.002105 | 0.04316 |
| *Enterococcus_sp._HMSC063H10* | PHES_1_ICT_0 | 4.332901 | 0.002117 | 0.04316 |
