# Supplementary figures and images for: Distinct gut microbial compositional and functional changes associated with impaired inhibitory control in patients with cirrhosis
Source: Gut Microbes. 2021 Aug 4;13(1):1953247. doi: 10.1080/19490976.2021.1953247 (PMC8344770; doi:10.1080/19490976.2021.1953247)

Figure S1: All Patients with Cirrhosis (N=97) underwent ICT and PHES

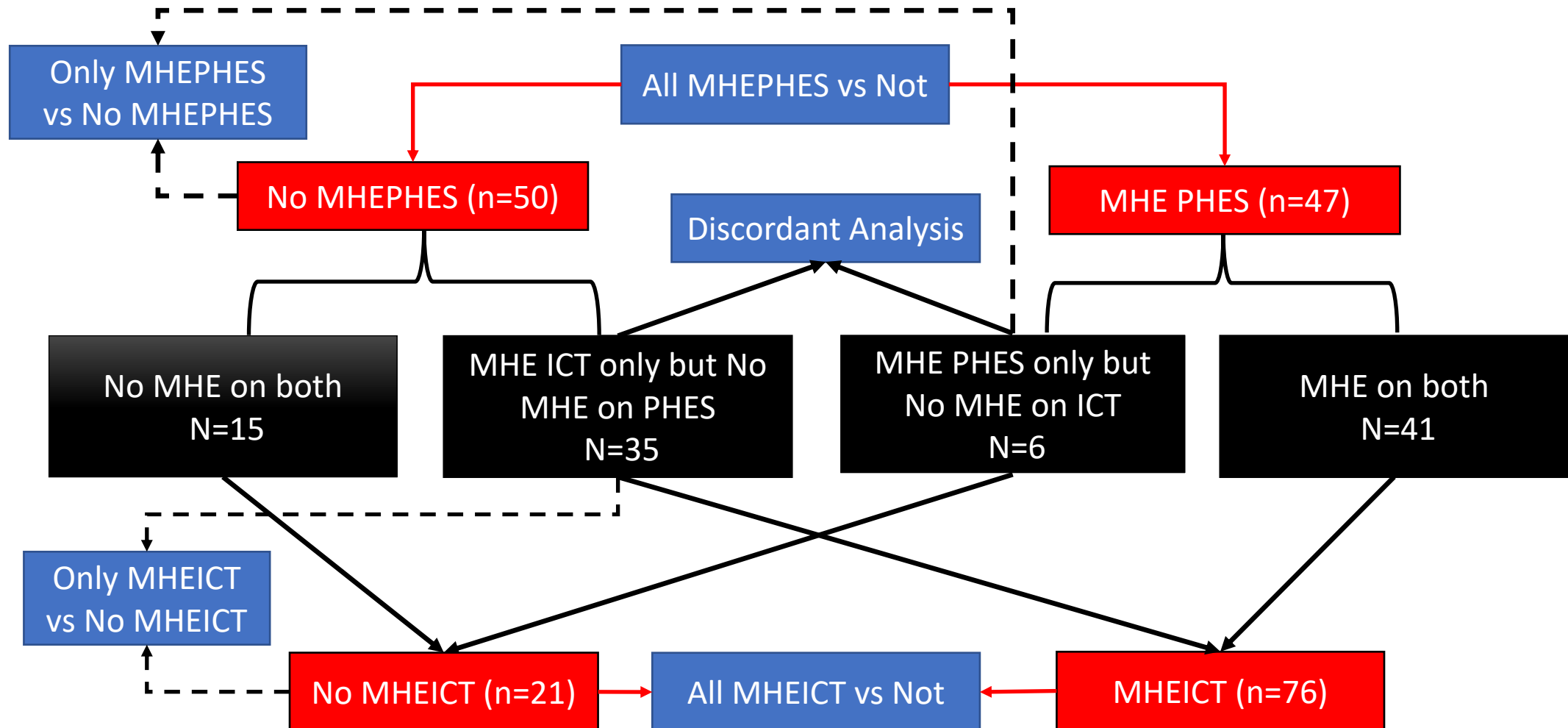

Supplement: Supplemental Material [file KGMI_A_1953247_SM3660.zip › supplementary/downloadFromZipFile.pdf]
